# A role for SOX9 in post-transcriptional processes: insights from the amphibian oocyte

**DOI:** 10.1038/s41598-018-25356-1

**Published:** 2018-05-08

**Authors:** M. Penrad-Mobayed, C. Perrin, D. L’Hôte, V. Contremoulins, J.-A. Lepesant, B. Boizet-Bonhoure, F. Poulat, X. Baudin, R. A. Veitia

**Affiliations:** 1 Institut Jacques Monod, CNRS UMR 7592, Université Paris-Diderot, Paris, France; 2 Unité de Biologie Fonctionnelle et Adaptative (BFA). CNRS UMR 8251, Université Paris Diderot, Paris, France; 3 Institut de Génétique Humaine (IGH), CNRS UMR 9002, Montpellier, France

## Abstract

*Sox9* is a member of the gene family of SOX transcription factors, which is highly conserved among vertebrates. It is involved in different developmental processes including gonadogenesis. In all amniote species examined thus far, *Sox9* is expressed in the Sertoli cells of the male gonad, suggesting an evolutionarily conserved role in testis development. However, in the anamniotes, fishes and amphibians, it is also expressed in the oocyte but the significance of such an expression remains to be elucidated. Here, we have investigated the nuclear localization of the SOX9 protein in the oocyte of three amphibian species, the urodelan *Pleurodeles waltl*, and two anurans, *Xenopus laevis* and *Xenopus tropicalis*. We demonstrate that SOX9 is associated with ribonucleoprotein (RNP) transcripts of lampbrush chromosomes in an RNA-dependent manner. This association can be visualized by Super-resolution Structured Illumination Microscopy (SIM). Our results suggest that SOX9, known to bind DNA, also carries an additional function in the posttranscriptional processes. We also discuss the significance of the acquisition or loss of *Sox9* expression in the oocyte during evolution at the transition between anamniotes and amniotes.

## Introduction

The SOX proteins (for “*SRY*-related HMG box”) are characterized by the presence of the high-mobility-group (HMG) DNA-binding domain. This domain of 79 amino-acids shares at least 50% identity with the HMG box of the mammalian male sex-determining SRY protein^1^. The SOX proteins constitute a large family of transcription factors (TFs), known to activate or repress gene expression and to be involved in a wide range of biological processes. On the basis of sequence similarity within the HMG box, mammalian SOX proteins have been divided into ten groups (A-J), each including one or several members. Orthologs of these genes have been found in different animal species. SOX TFs influence local chromatin structure through their capacity to bend DNA. In most cases, the HMG box of SOX proteins binds to target DNA sequences and a consensus binding site [(A/T)(A/T)CAA(A/T)G] has been defined^2^. Regions outside of the HMG domain play also a role in DNA-binding. However, the cell-specific action of SOX proteins is mainly due to their interaction with partners to form stable transcription complexes (for recent reviews see^3–9^). Besides their activity as TFs, several studies have highlighted a potential role of SOX proteins in RNA processing^10–15^. In addition, a direct interaction with RNA has been shown for several of them. In *Drosophila*, the Dichaete Sox protein binds to synthetic target RNAs in a sequence-specific manner^16^. SOX2 physically interacts with the long noncoding RNA rhabdomyosarcoma 2-associated transcript (RMST) and this interaction is necessary for the activation neural genes^17^.

The SOX9 protein belongs to the group E that also includes SOX8 and SOX10. SOX9 is highly conserved among vertebrate species and is involved in multiple organogenetic processes, such as chondrogenesis, neurogenesis and gonadogenesis^18^. The *Sox9* gene is the major target of *Sry*, the master testis-determining gene in mammals, located on the Y chromosome. In mammals, where it has been most extensively studied, *Sox9* has been shown to play a critical role in male sex determination and testis differentiation. Its mutation or deletion leads to 46,XY Disorders of Sex Development (formerly known as male-to-female sex reversal) in humans and mice^19^. During embryogenesis, the SOX9 protein is detected in the cytoplasm of somatic cells in male and female gonads. At the onset of testicular differentiation, SOX9 moves to the nucleus of Sertoli cells precursors where it is detected up to the adult stage. In contrast, the expression of SOX9 is downregulated during ovarian differentiation^20–22^. Nuclear translocation of SOX9 constitutes a key event in the differentiation of the male gonad and its impairement leads to 46,XY DSD (male-to-female sex-reversed phenotype). In contrast, experimentally-forced nuclear localization of SOX9 in the XX gonad leads to a female-to-male sex reversal^23–25^. Recently, genetic networks directly regulated by SOX9 in the mammalian foetal testis were identified and a direct or indirect role in the modulation of pre-mRNA splicing of several of its target genes was shown^15^.

The function of the *Sox9* gene during gonadogenesis has been less well investigated in other vertebrate species. In birds^26^ and reptiles^27,28^, it is expressed in a male-specific manner, whereas in fishes^29–35^ and amphibians^36–40^ it is expressed in both the male and female gonads. Of note, the majority of these investigations focused on the expression of the *Sox9* gene only at the RNA level.

We have previously shown that the expression of the SOX9 protein in the amphibian *Xenopus tropicalis* greatly differs between the male and female gonads^38^. In the testis, SOX9 is localized in the nuclei of Sertoli-like somatic cells, similarly to what has been observed in mammals. In contrast, it is localized in the germ cells of the female gonad. In the juvenile female gonad, SOX9 is exclusively detected in oocyte cytoplasm. Surprisingly, in the adult gonad, it is detected in both the cytoplasm and the nucleus (also called germinal vesicle or GV). This unexpected observation of a translocation of SOX9 into the GV prompted us to check whether it also takes place in other amphibians and to investigate its functional significance.

We took advantage of the remarkable structure of the giant chromosomes of the amphibian oocyte, the lampbrush chromosomes (LBCs), to analyze the sub-nuclear localization of the SOX9 protein on LBC spreads of three amphibian species, the anurans *X. tropicalis* and *X. laevis*, and the urodelan *Pleurodeles waltl*. Similarly to polytene chromosomes of insects, amphibian LBCs offer the unique possibility to use light microscopy to visualize directly on nuclear spreads the condensed and transcribed uncondensed chromatin regions at the whole genome level and to identify associated proteins^41–50^ (http://projects.exeter.ac.uk/lampbrush/). In this study, we provide evidence for the association of the SOX9 protein with RNP transcripts in an RNA-dependent manner and we show that this association can be visualized by super-resolution imaging. Our findings suggest a role for SOX9 in post-transcriptional processes.

## Results

### The SOX9 protein is detected in the nuclear and cytoplasmic extracts from *X. tropicalis*, *X. laevis* and *P. waltl* oocytes

**Figure 1:**
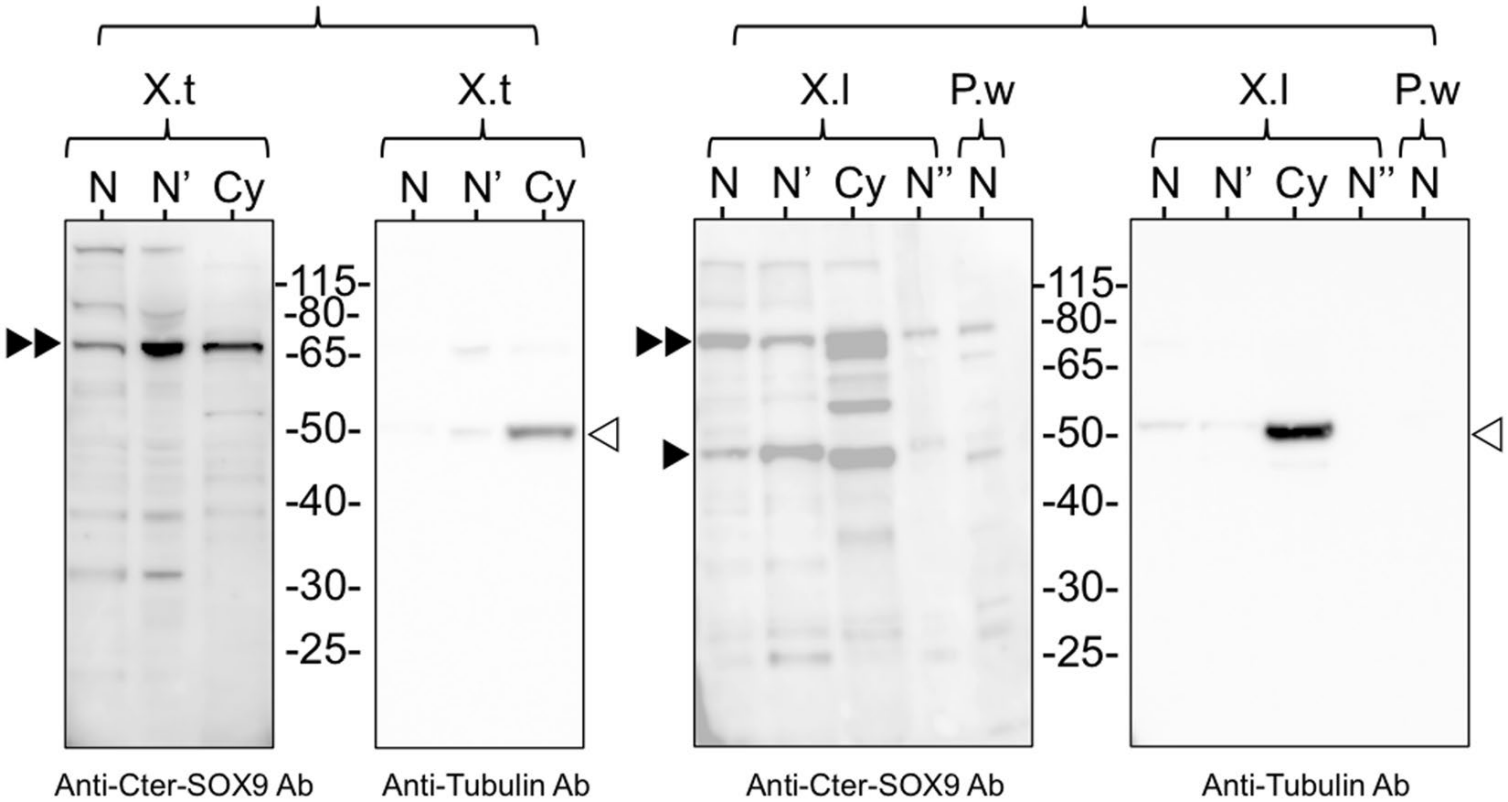
Immunodetection of SOX9 in the oocyte of *X. tropicalis*, *X. laevis and P. walt*. Western blot analysis of SOX9 in nuclear and cytoplasmic extracts using the polyclonal anti-Cter-SOX9 antibody and the monoclonal anti-tubulin antibody. (N) corresponds to 25 GVs from *X. tropicalis* (X.t), 20 from *X. laevis* (X.l) and 15 from *P. waltl* (P.w). (N’) nuclear extract without nuclear envelope. (N”) 15 GVs from *X. laevis*. (Cy) cytoplasms from 10*X*. *tropicalis* oocytes and from 5*X*. *laevis* oocytes. The anti-Cter- SOX9 antibody recognized a major polypeptide around 68-kDa in the nuclear (N and N’) and cytoplasmic (Cy) extracts of the three species (black double arrowheads) and an additional one at 48 kDa (black arrowhead). The control anti-tubulin antibody recognized a 50-kDa polypeptide only in the cytoplasmic extracts (empty arrowheads).

SOX9 nuclear sub-localization was studied using two polyclonal antibodies directed against the N-ter and C-ter regions of the human protein. Although these regions present a high degree of amino acid identity with those of *X. tropicalis*, *X. laevis* and *P. waltl* proteins (Fig. S1), we first confirmed that the antibodies cross-reacted with the exogenous *X. tropicalis* SOX9 protein expressed in mammalian cells after transfection with a plasmid vector (see Materials and Methods, Fig. S2). We then investigated by western blot the presence of the amphibian SOX9, in both nuclear and cytoplasmic extracts of *X. tropicalis*, *X. laevis* and *P. waltl* oocytes. Envelope-free nuclear extracts were also analyzed to exclude that the detection of SOX9 in the GV was due to a contamination of the nuclear extracts by a small amount of cytoplasm remaining associated with the nuclear envelope. Alpha-tubulin (detected by a monoclonal antibody) was used as a cytoplasmic control. As expected, the two polyclonal anti-SOX9 antibodies recognized one polypeptide of nearly the same apparent molecular weight at around 68-kDa in *X. tropicalis*, *X. laevis* and *P. waltl* nuclear and cytoplasmic extracts (Fig. 1, and Fig. S3), whereas the mouse mAb against alpha-tubulin detected one polypeptide of the expected size (52 kDa) in cytoplasmic extracts only. These results confirmed the presence of SOX9 in the amphibian oocyte and its nuclear localization. They are in contrast to those of Dumond *et al*.^37^ who did not detect SOX9 in the GV of *P. waltl* ovocyte using the same Nter-SOX9 Ab antibody^51^. One possible explanation for this discrepancy is that they used the anti-SOX9 antibody at a higher dilution (1:1000 instead of 1:400 used here).

### The SOX9 protein binds to LBCs lateral loops and nuclear bodies in the three amphibian species

**Figure 2:**
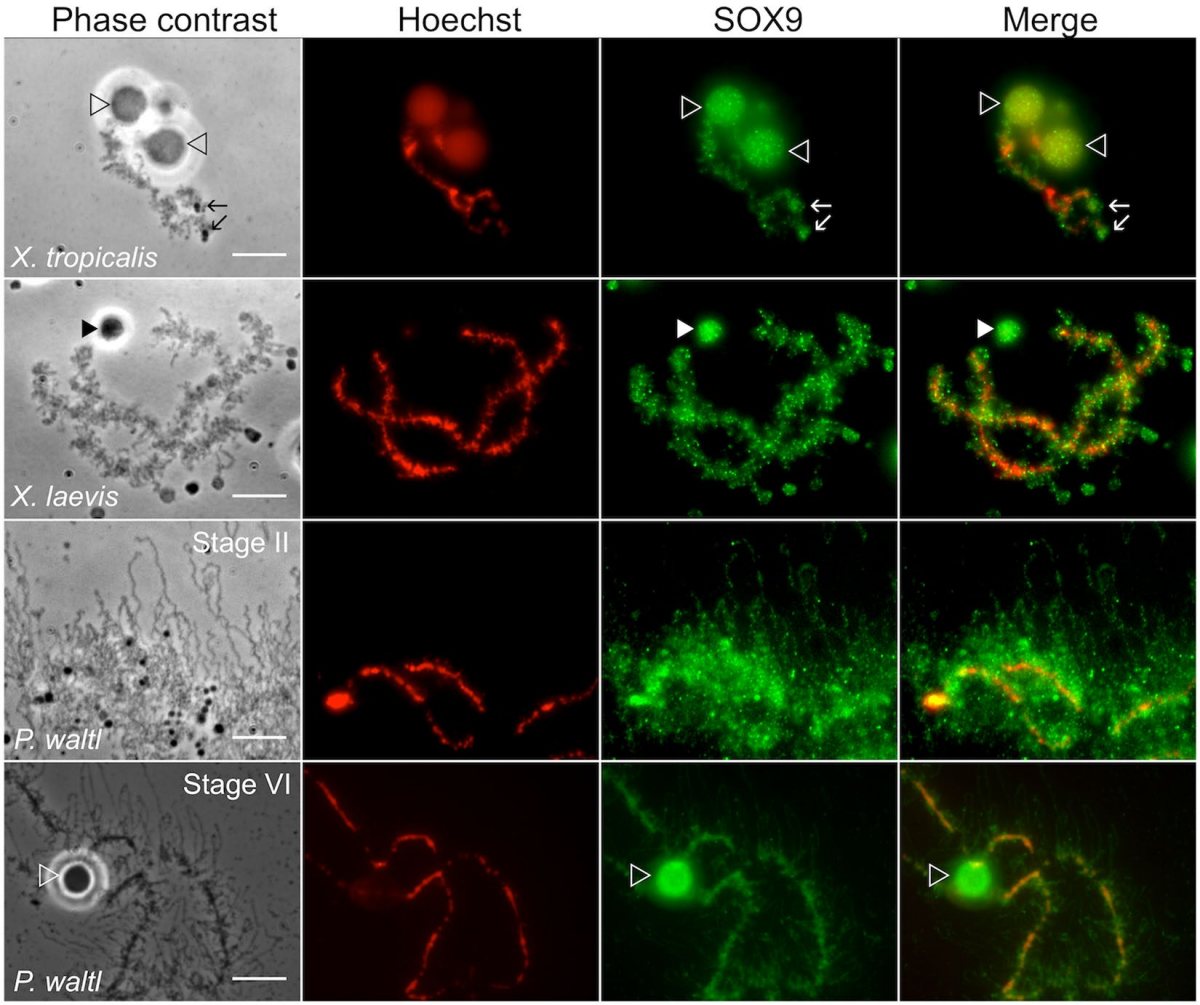
Subnuclear localization of the SOX9 protein in the GV of *X. tropicalis*, *X. laevis* and *P. waltl*. Fluorescent and corresponding phase contrast micrographs of nuclear spreads showing one LBC of *X. tropicalis* and *X. laevis* from stage V-VI oocyte, and part of a *P. waltl* LBC from stages II and V-VI oocyte. Nuclear spreads were immunostained with the anti-Cter-SOX9 antibody (green, Alexa 488 IgG) and counterstained with Hoechst (red). The Hoechst dye stained the chromosome DNA axis and not that of the decondensed lateral loops. The antibody labeled the LBCs, and the nuclear bodies either attached to them (empty arrowheads) or free in the nucleoplasm (full arrowheads). Note that the immunostaining of lateral loops was very clear in *P. waltl* LBCs from stage II oocyte, because of their extension and density. The intensity of fluorescence in the different experiments was normalized with respect to their relevant controls with the secondary antibody (tagged with Alexa fluor 488) alone. Wide field Leica microscope. Scale bar for all micrographs: 10 μm.

The meiotic lampbrush chromosomes are associated in bivalent pairs. Their axes comprise a succession of highly condensed chromatin regions, the chromomeres, from which lateral loops unfold in opposite directions. The chromomeres constitute the main chromosome axis and can be visualized by DAPI or Hoechst staining. The lateral loops are clearly visible by light microscopy. They serve as template for RNA polymerase II-dependent transcription and comprise one or more transcription units. The nascent RNA transcripts associate with proteins to form the ribonucleoprotein (RNP) matrix whose thickness shows a marked polarity along its length according to the putative direction of transcription. The decondensed transcribed DNA axis of lateral loops is itself neither visible by light microscopy nor after DAPI or Hoechst staining (for review, see:^52–54^). As shown in Fig. 2, the sizes of the LBCs and their lateral loops differ between the three amphibians with a direct correlation with genome size as reported for many amphibian species^55^. *P. waltl* (C value: 19,3 pg) LBCs are larger than those of *X. laevis* (C value: 3 pg), which are in turn larger than those of *X. tropicalis* (C value: 1.5)^56^. The mean size of the lateral loops in *P. waltl* is 50 μm while it is around 5–10 μm in *X. laevis*. Furthermore, for a given species, the size of the LBCs and the number and size of their lateral loops, are also directly related to their transcriptional activity. Consistently, the loops are more developed at early oocyte stages, when transcription is intense, than at stage VI when transcription is almost arrested.

When applied to nuclear spreads of *X. tropicalis, X. laevis* or *P.waltl* oocytes, both the anti N-ter and anti C-ter SOX9 antibodies strongly labelled the lateral loops of LBCs and nuclear bodies, which were either free or attached to the LBCs, as it is the case for the Histone Locus Body (HLB) appearing as a sphere bound to chromosome XI of *P. waltl* (Fig. 2 and Fig. S4-A). Some of the free bodies corresponded to the Cajal bodies (CBs) because they were co-stained for Coilin, one of its major components^57,58^ (Fig. S4-B). The immunostaining of lateral loops was best displayed in the case *P. waltl*, particularly at an early oocyte stage, when they were large and well extended from the chromosome axis.

### Confirmation of the SOX9 immunostaining pattern

**Figure 3:**
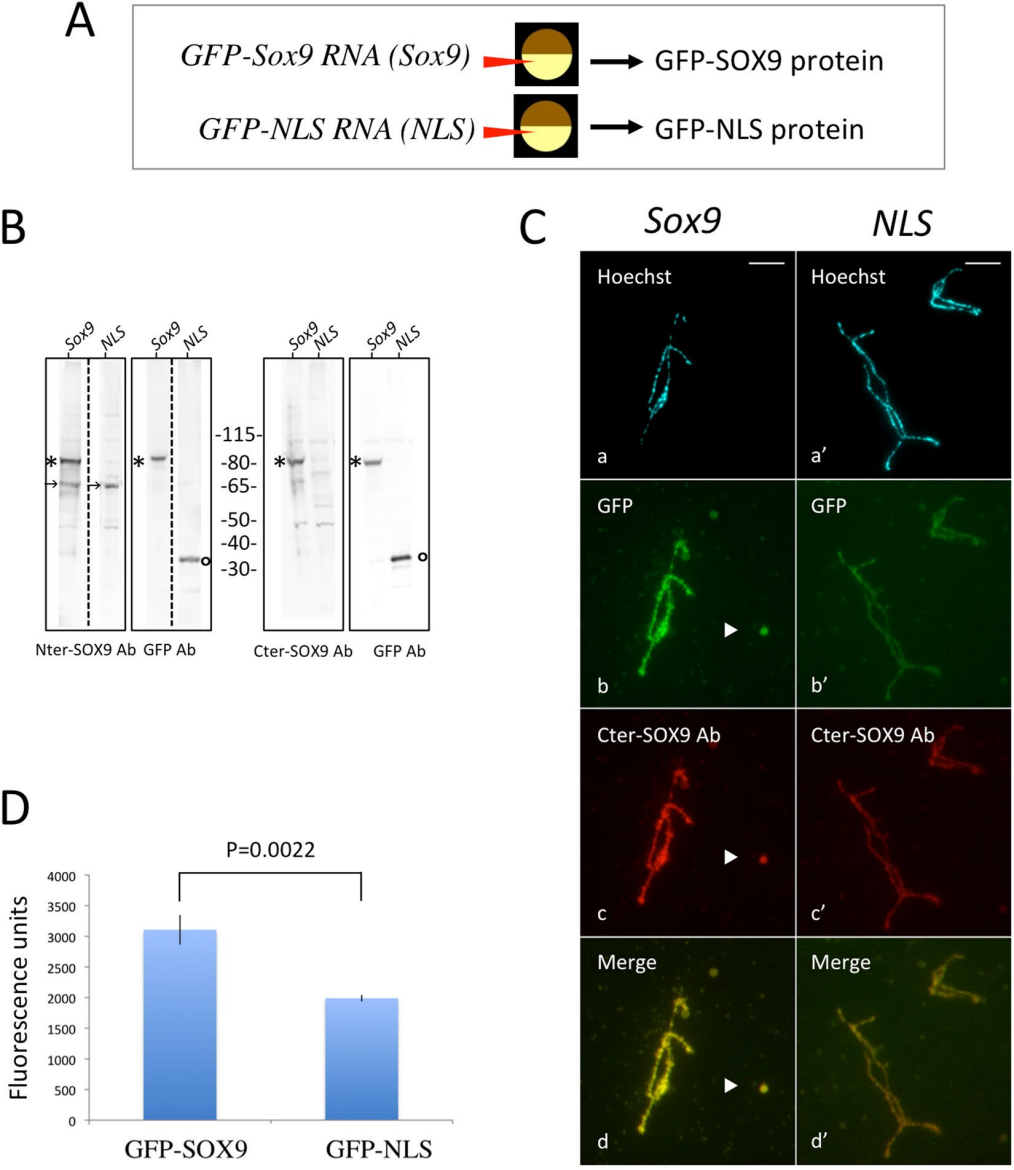
Targeting of the newly-synthetized Xt-SOX9-GFP protein to LBCs and nuclear bodies. (**A**) Capped, *in vitro*-synthetized transcripts from the *pcDNA Xt-sox9- CT-GFP (Sox9)* vector, or the control *pcDNA NLS-CT-GFP (NLS)* vector, were injected into the cytoplasm of stage IV-V oocytes of *X. laevis*. The expressed proteins (Xt-SOX9-GFP or NLS-GFP) were detected 18 hours later, either directly on nuclear spreads by fluorescence microscopy or indirectly on immunoblots using antibodies against the SOX9 or GFP proteins. (**B**) Immunoblots of nuclear extracts from injected oocytes with the *sox9-CT-GFP* (*Sox9*) or *NLS-CT-GFP* (*NLS*) transcripts. Each lane corresponds to 15 GVs. kDa: molecular weight markers. A polypeptide (*) around 80 kDa detected with the anti-Nter or Cter- SOX9 antibodies and the anti-GFP antibody in the *Sox9-CT-GFP* injected oocytes corresponded to Sox9-GFP protein. In the *NLS-CT-GFP* injected oocytes, the anti-GFP antibody detected a 30 kDa polypeptide (°) corresponding the NLS-GFP protein. The polypeptide of ca. 68 kDa (arrows) corresponds to the endogenous SOX9 protein. The dashed lines separate the lanes which were cut from the original immunoblots shown in the supplementary figure S5. (**C**) (b, b’) Direct detection of GFP-SOX9 and GFP-NLS proteins on nuclear spreads from the injected oocytes. The GFP-SOX9 protein targeted the LBCs and the free nuclear bodies (arrowhead). A much lower level of GFP-NLS was detected on the LBCs. (c, c’) immunostaining of the nuclear spreads using the anti-Cter-SOX9 antibody. (d, d’) merged images (b, b’ and c, c’) indicating that the staining pattern of the Xt-SOX9-GFP was similar to that using the anti-Cter-SOX9 antibody. Wide field Leica microscope. Scale bar for all micrographs: 10 μm. (**D**) Quantification of GFP fluorescence density on LBCs with the Otsu’s method. GFP-fluorescence density over the GFP-SOX9 LBCs was significantly higher than that of the control GFP-NLS LBCs (Student’s test, p = 0.002).

To confirm the immunostaining pattern of the nuclear spreads observed using the anti-SOX9 antibodies, the sub-nuclear localization of an exogenous GFP-tagged SOX9 protein expressed from RNA-injected oocytes was analyzed. *In vitro*-synthesized capped transcripts coding for either a GFP-tagged *Xt-* SOX9 (Xt-SOX9-GFP) protein or the NLS-GFP protein as a control, were injected into the cytoplasm of stage V-VI *X. laevis* oocytes (Fig. 3A). After 24 h of incubation, the Xt-SOX9-GFP and NLS-GFP proteins were detected in the nuclear extracts by western blotting using antibodies against SOX9 and GFP proteins at the expected molecular weights (Fig. 3B and Fig. S5). In parallel, the targeting behavior of these exogenous proteins was analyzed on nuclear spreads by fluorescence microscopy. The Xt-SOX9-GFP protein targets the LBCs and nuclear bodies (Fig. 3C-b). This staining pattern was similar to the one obtained with the anti-SOX9 antibodies (Fig. 3Cc-d). Because of a possible non-specific binding of the NLS-GFP protein to the chromosomes, we quantified the fluorescence intensity using the Otsu’s method by first devising appropriate masks on the Hoechst images to define a region of interest (ROI) (Materials and Methods, Fig. S6). The fluorescence intensity of the Xt-SOX9-GFP protein on the LBCs was much higher than for NLS-GFP (Fig. 3D). Altogether, these and the immunostaning results show that SOX9 binds to the lateral loops and nuclear bodies.

### SOX9 does not bind the chromosome axis of the LBCs

**Figure 4:**
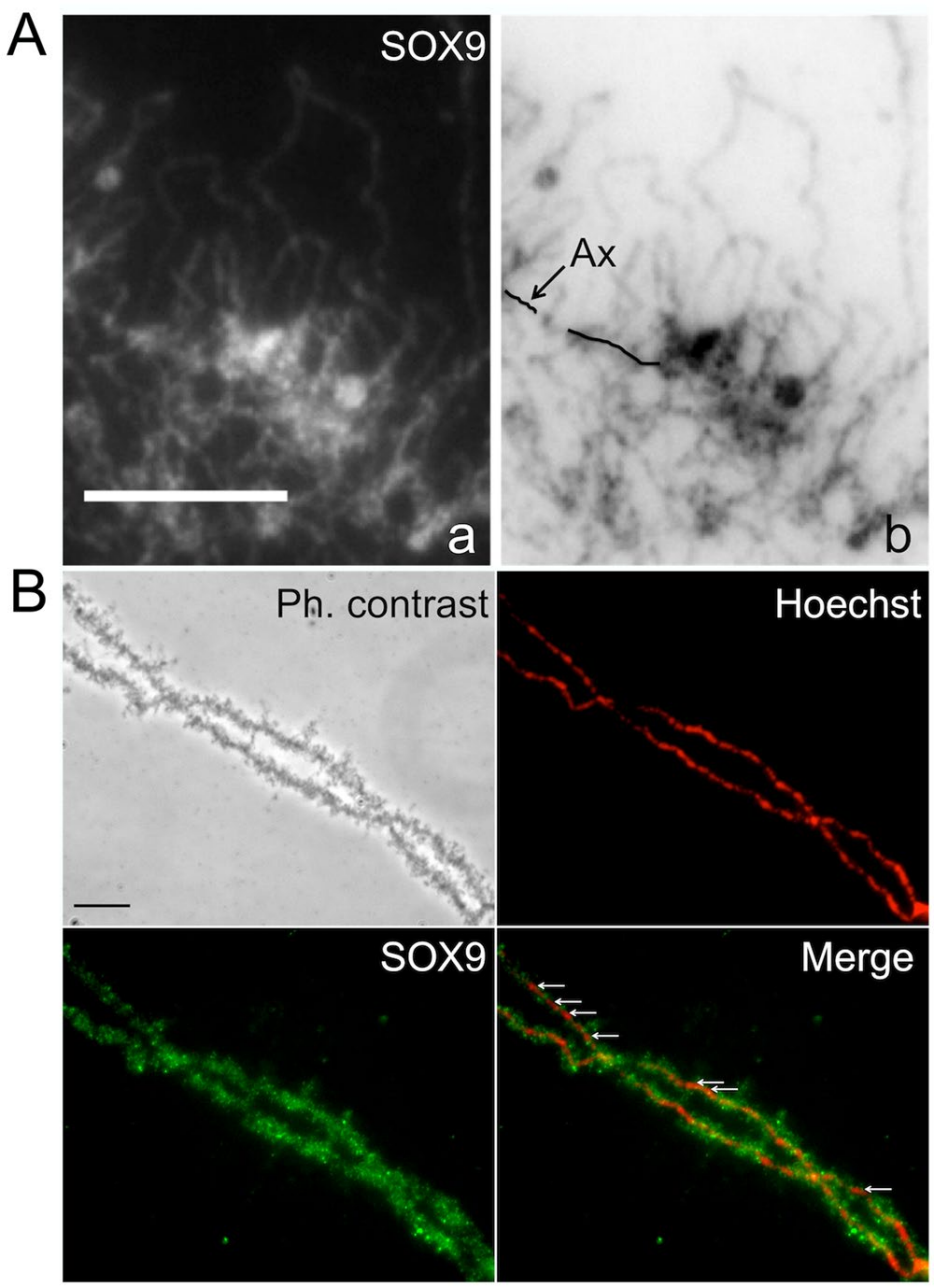
SOX9 protein does not bind the chromosome axis of the LBCs. Immunostaining of *P. waltl* nuclear spreads using the anti-Cter-SOX9 antibody (green, Alexa 488 IgG) and counterstained with Hoechst (red). (**A**) Fluorescent micrograph (a) and its corresponding negative (b) showing a strong SOX9 immunostaining detected in the close vicinity of the chromosome axis over the thickest region of the lateral loops (**B**) Nuclear spreads from an oocyte at the end of stage VI. The SOX9 protein was not detected at the level of the chromosome axis where it was devoid of lateral loops (white arrows, merge panel). Scale bar for all micrographs: 10 μm.

**Figure 5:**
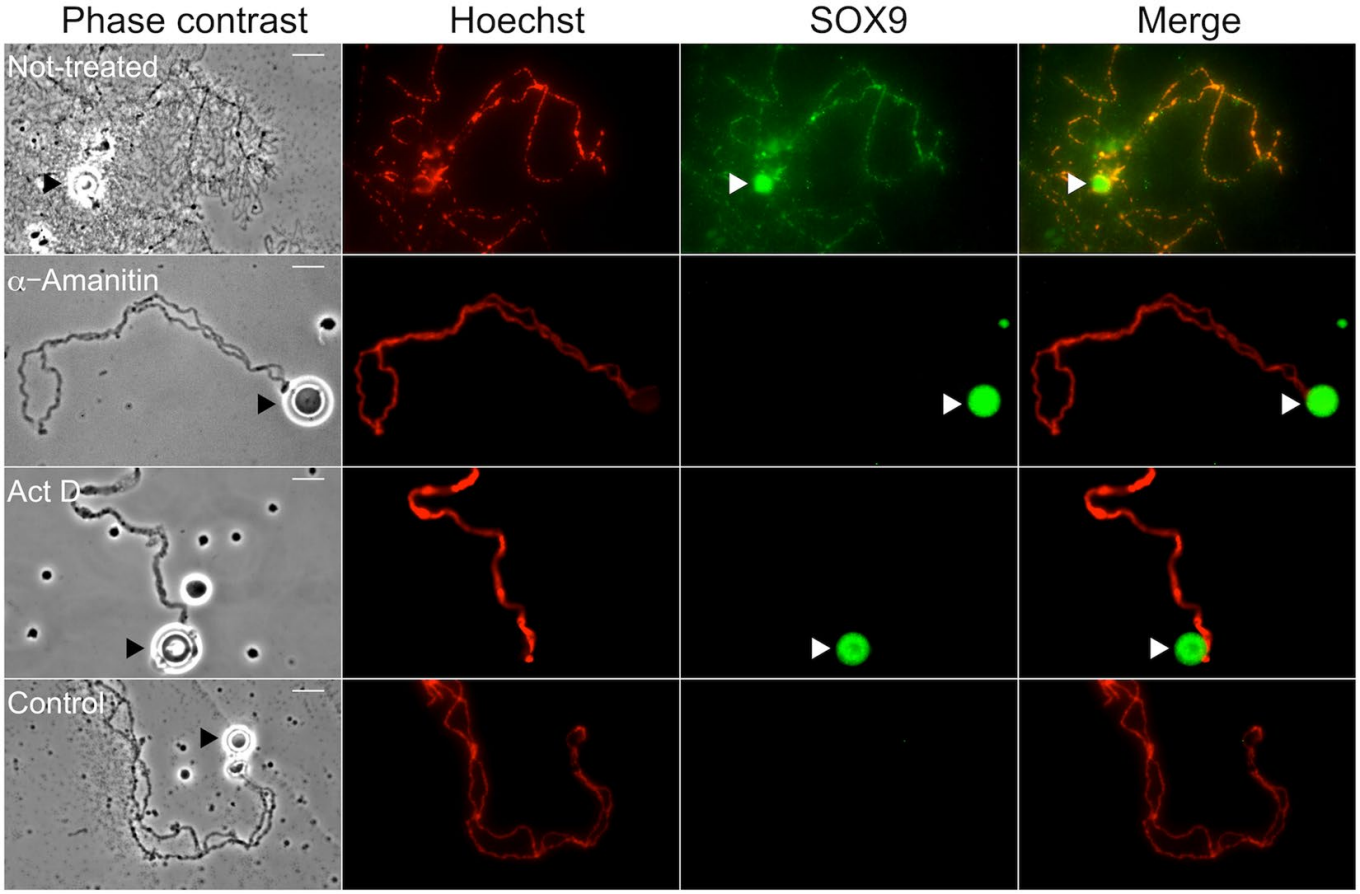
Immunostaining of SOX9 after inhibition of transcription. Oocytes of *P. waltl* were treated or not with α-Amanitin or Actinomycin D (Act D). Phase contrast and corresponding fluorescent micrographs of nuclear spreads that were stained with the anti-Cter SOX9 antibody (green, Alexa 488 IgG) and counterstained with Hoechst (red) to show the chromosome axis. In contrast to the LBCs from the not-treated oocytes, those from the oocytes incubated with α-amanitin or Act D were devoid of their lateral loops and and the chromosome axis lacked SOX9 staining. Note that the spheres (arrowheads) known to be storage sites for transcription and post-transcription factors were strongly stained. The intensity of fluorescence in the different experiments was normalized with respect to their relevant controls with the secondary antibodies alone. Wide field Leica microscope. Scale bar for all micrographs: 15 μm.

The strong immunostaining which can be observed over the chromosome axis with the SOX9 antibodies as shown in Figs 2 and 3, raised the question as to whether SOX9 actually binds to the condensed chromatin of the chromomeres, which constitute the visible part of the chromosome axis and from which the decondensed chromatin axes of the lateral loops unfold. Three lines of evidence suggest that SOX9 does not bind to the chromomeres. First, in some preparations where the lateral loops were particularly well extended from the chromosome axis and exhibited an obvious polarity as shown in Fig. 4A, a strong SOX9 immunostaining was detected in the close vicinity of the chromomeres over the thickest region of the lateral loops corresponding to the presumed termination of transcription. This pattern may explain why the SOX9 immunostaining appeared to overlap with the chromosome axis in less resolved preparations. Second, SOX9 immunostaining was not detected at the level of the chromomeres when they were devoid of lateral loops as seen with LBCs from oocytes at the end of stage VI (Fig. 4B). A third argument was brought by transcription inhibition experiments using alpha-amanitin or actinomycin D. Although these two drugs act differently, the former being a DNA intercalator blocking the progression of the three RNA polymerases, and the later a highly selective inhibitor of RNA polymerases II and III (for a review see^59^), their effect on LBCs was the same. As previously described^60^, after incubation of the oocytes with these drugs, transcription was arrested, the nascent RNP transcripts were released, the lateral loops underwent retraction and the chromosomes being devoid of their lateral loops became extremely condensed. Under these conditions, no labelling of the chromosome axis with the SOX9 antibodies was observed, although it was still detected over free Cajal Bodies or the nuclear bodies associated with them (Fig. 5).

### SOX9 is associated with the RNP matrix of lateral loops in an RNA-dependent manner

**Figure 6:**
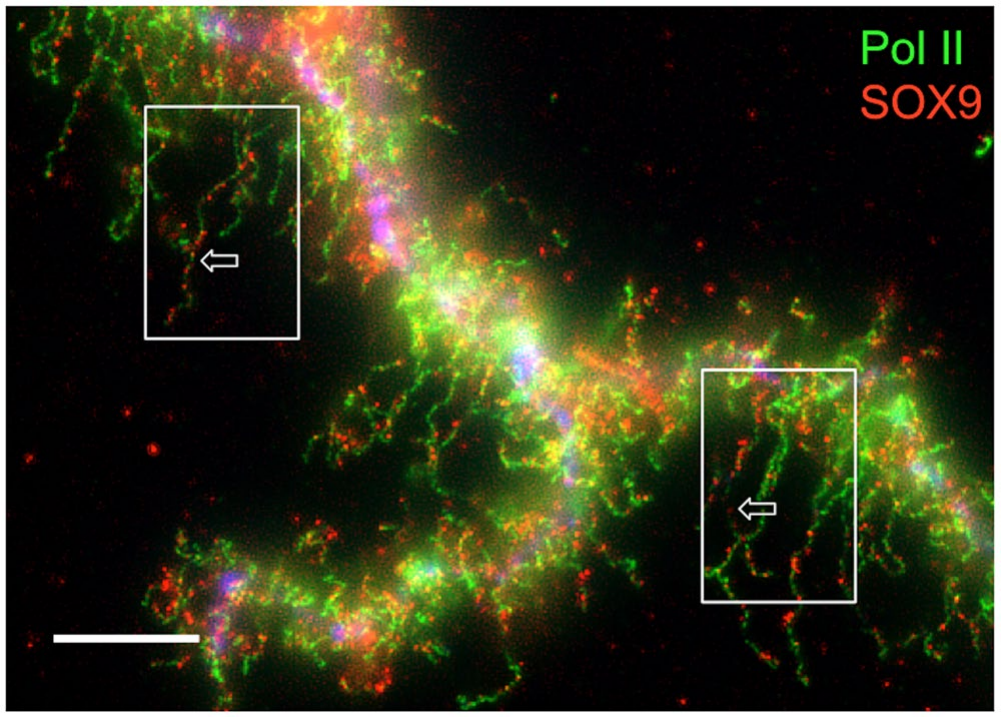
Immunostaining of Pol II and SOX9 in *P. waltl* LBCs. Nuclear spreads were immunostained for SOX9 with the anti-Cter-SOX9 antibody (red, Alexa 568 IgG) and for Pol II with the mAbH14 (green, Alexa 488 IgM). Pol II immunostaining was continuous over the loop axis while that of SOX9 was concentrated on granules distributed above the loop axis. This double immunostaining pattern was particularly visible on the two lateral loops indicated by arrows in the boxed areas. The intensity of fluorescence in this experiment was normalized with respect to its relevant controls (i.e., secondary antibodies alone). The image was deconvoluted (see Figure S7 for the non deconvoluted image). Wide field Leica microscope. Scale bar: 10 μm.

**Figure 7:**
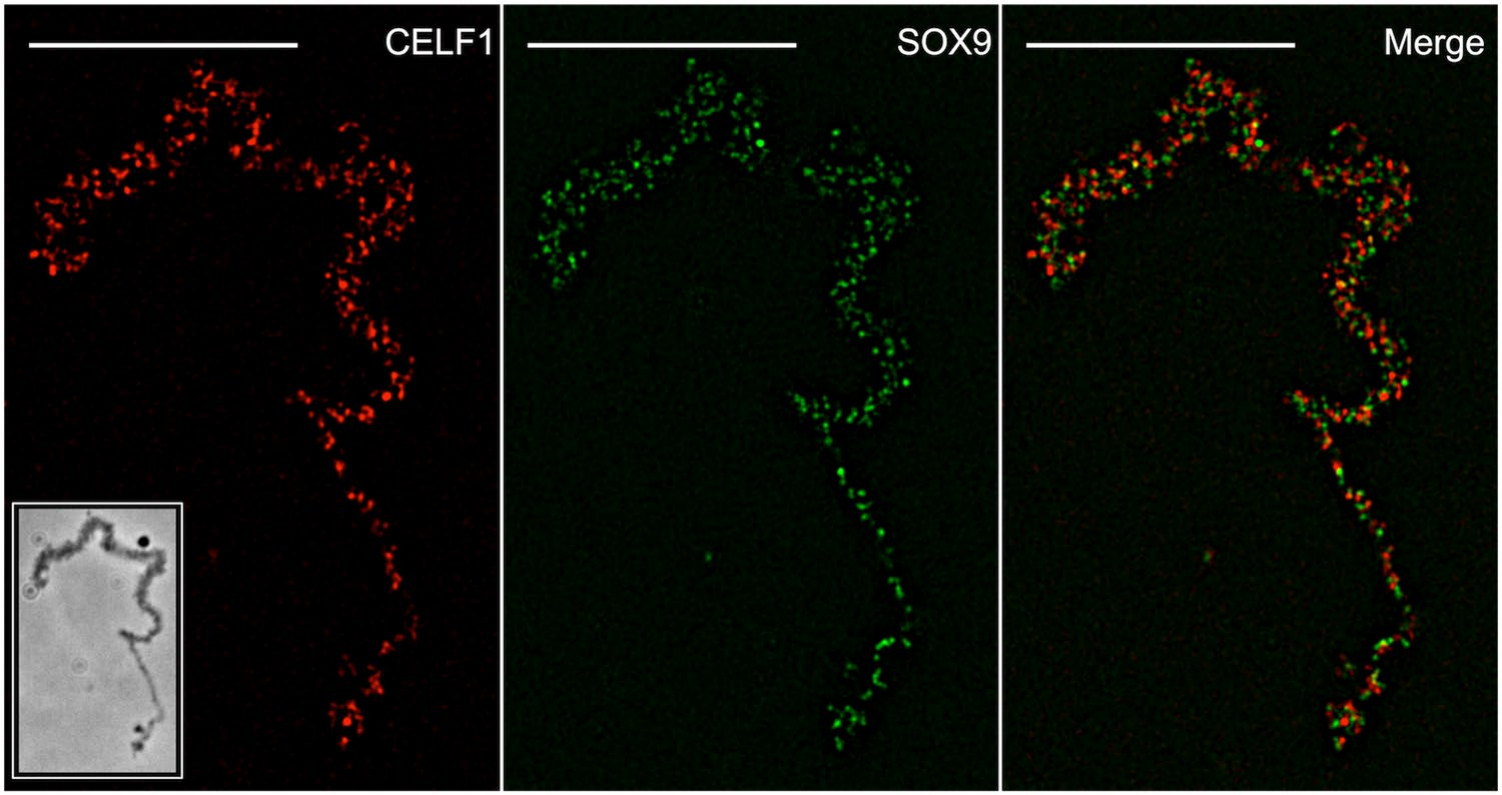
Super-resolution images of a *P. waltl* lateral loop immunostained for CELF1 [mAb3B1 (red, Alexa 568 IgG)] and SOX9 [anti-Cter-SOX9 antibody (green, Alexa 488 IgG)]. The RNP matrix exhibited a pattern of granules immunostained for SOX9 and CELF1. The merged panel showed that the SOX9 and CELF1 granules did not colocalize. The intensity of fluorescence in the different experiments was normalized with respect to their relevant controls with the secondary antibodies alone. Structured Illumination Microscopy (SIM). The image in the boxed area corresponded to the phase contrast image obtained from Wide field Leica microscopy. Scale bar: 10 μm.

We performed a detailed analysis of the SOX9 immunostaining pattern of lateral loops to determine whether the protein binds to the transcribed DNA axis of the lateral loops, the associated RNPs transcripts or both. Because Hoechst staining was not strong enough to detect the decondensed transcribing chromatin axis of the lateral loops, we carried out co-immunostainings using the SOX9 antibody together with an antibody (mAb H14) directed against phosphorylated RNA Polymerase II (referred to as Pol II), which was previously reported to label the DNA axis of lateral loops undergoing transcription^53,54^. As expected, both antibodies stained the lateral loops (Fig. 6 and Fig. S7). However, while the Pol II immunostaining appeared continuous over the axis of the lateral loops, the SOX9 immunostaining showed a punctate pattern with granules distributed above the axis of the loops. This suggests that SOX9 is associated with the RNP matrix rather than with the chromatin axis of lateral loops. To confirm this result, co-immunostaining experiments were performed using antibodies directed against CELF1 and SR proteins, previously reported to be associated with the nascent RNA transcripts^44,47^. CELF1 is a multifunctional RNA-binding protein involved in the regulation of alternative splicing^61^. Fluorescence microscopy experiments have shown that CELF1 is able to bind to a subset of lateral loops in several amphibian species, Xe*nopus laevis*, *Triturus vulgaris* and Axolotl^44^. Its binding to RNP transcripts was confirmed by super-resolution imaging using Spectral Position Determination Microscopy (SPDM) in *Notophthalmus viridescens*^47^. In *P. waltl* we also detected it on a few lateral loops (Fig. S8). The pattern of co-immunostaining performed using antibodies against SOX9 and CELF1 was analyzed using Super-resolution Structured Illumination Microscopy (SIM). The superresolution microscopy images revealed a pattern of granules immunostained with either SOX9 or CELF1 antibodies, which corresponded to packed RNP transcripts (Fig. 7). The average size of these particles as determined using the spot module of the IMARIS software (Bitplane, version 8.4) was similar for those associated with SOX9 (0.192 µm, standard deviation: 0.035) or CELF1 (0. 196 µm, standard deviation: 0.038). The Pearson’s correlation coefficient of the intensities of the fluorescence signal calculated using the Coloc2 plugin of the Image J program, was negative (R = −0.65, p < 0.0001)), excluding a colocalization of SOX9 and CELF1 on the same granules, i.e, on the same RNA-protein complexes.

**Figure 8:**
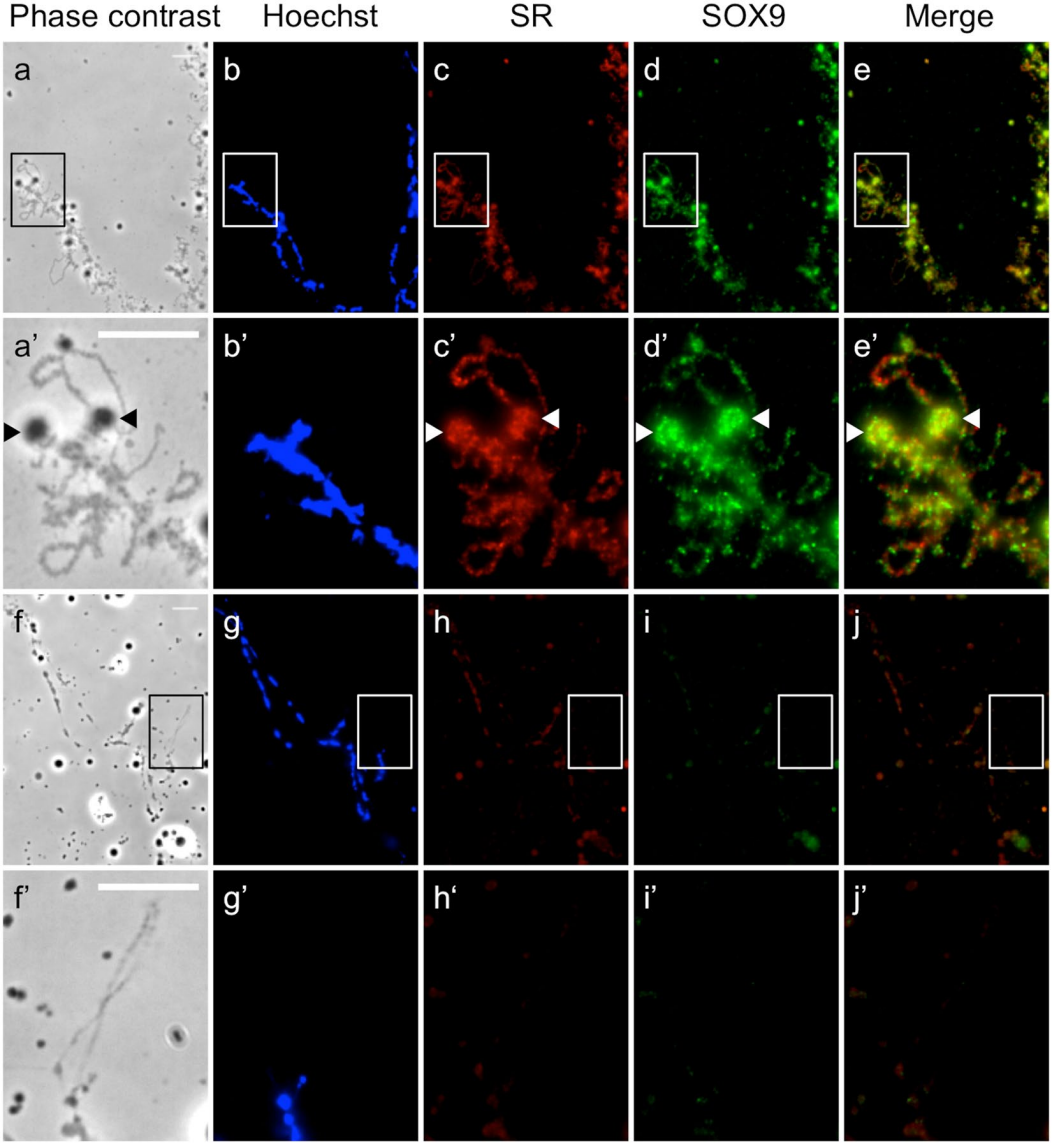
Localization of SOX9 to the matrix of lateral loops is RNA-dependent. Nuclear spreads of *X. laevis* were immunostained for SOX9 [anti-Cter-SOX9 antibody (green, Alexa 488 IgG)] and SR proteins [mAb1H4 (red, Alexa 568 IgG)]. (**a**–**e**’) LBC from untreated nuclear spreads. (**f**–**j**’) LBC from nuclear spread digested with RNase A before immunostaining. (**a**’–**e**’) Enlarged images of the regions marked by white boxes (**a–e**) show several lateral loops immunostained with SOX9 and SR antibodies. The arrowheads point to terminal granules. (**f**’–**j**’) enlarged images of the regions marked by white boxes (**f–j**) show one lateral loop not stained with SOX9 and SR antibodies after digestion with Rnase A. The intensity of fluorescence in the different experiments was normalized with respect to their relevant controls (the secondary antibodies only). Wide field Leica microscopy. Scale bar: 10 μm.

SR proteins belong to a conserved family of pre-mRNA splicing factors that are associated with the RNA transcripts of lateral loops^62,63^. Co-immunostaining on LBCs of *Xenopus laevis* using the anti C-ter SOX9 antibody and the monoclonal mAb1H4 antibody directed against SR proteins of *X. laevis* showed, as expected, that the two antibodies stained almost all the lateral loops (Fig. 8a-e’). In order to determine whether the binding of SOX9 to lateral loops was RNA-dependent, preparations of *X. laevis* LBCs were treated with RNAse A before co-immunostaining with the SOX9 antibody or the monoclonal mAB1H4. No immunofluorescence was detected over the lateral loops when LBCs preparations were treated with RNAse (Fig. 8f-j’). This clearly indicated that the association of SOX9 to the RNP matrix was mediated directly or indirectly by RNA.

## Discussion

The aim of this study was to establish whether the nuclear localization of SOX9, previously reported in the oocyte of *X. tropicalis*, took also place in other amphibian species such as *X. laevis* and *P.waltl*, as a way to get insights into its functional role during oogenesis. Using two different approaches relying either on SOX9 antibodies or on the production of exogenous GFP-tagged SOX9 protein, we have provided evidence for the nuclear localization of SOX9 and its specific association with the nascent RNP matrix of lateral loops in all three amphibian species. Because no SOX9 immunostaining was found on LBCs devoid of lateral loops, either at the late stage VI of oocyte development or after inhibition of transcription by either actinomycin D or alpha-amanitin, it can be excluded that SOX9 binds to the non-transcribed chromatin of the chromosome axis.

As mentioned above, lateral loops are sites of intense RNA synthesis and display a high density of RNA polymerase II complexes. The nascent RNA transcripts associate with proteins that are involved in their packaging, processing, editing, degradation and/or transport. The labelling pattern resulting from the co-immunostaining with antibodies directed against SOX9 and Pol II strongly suggests that the former does not associate with the decondensed DNA axis of lateral loops undergoing transcription but rather with the resulting RNP transcripts. The fact that the association of SOX9 with the RNP matrix is RNA-dependent supports this conclusion. The super resolution microscopy data clearly show that SOX9 exhibits the same granular fluorescence pattern as CELF1 which is involved in the regulation of alternative splicing and known to be associated with the nascent RNP transcripts. SOX9 was also shown to localize to the Cajal and HLBs that are known storage sites for transcriptional factors and those involved in post-transcriptional processes^57,58^. Altogether these data lead us to propose that the association of SOX9 with the lateral loops results from its binding to the RNA transcripts either directly or indirectly through protein-protein interactions. This raises the question as to whether the amphibian SOX9 protein has a post-transcriptional function. Several studies carried out in different cellular systems have shown that SOX9 proteins could have a role in splicing regulation. Hata *et al*.^12^ have demonstrated that SOX9 strongly interacts with the paraspeckle protein p54^nrb^ that regulates both transcription and splicing of the *Col2a1* mRNA in the chondrogenic cell line ATDC5. More recently, Rahmoun *et al*.^15^ have shown in mammalian fœtal testes that SOX9 not only regulates the transcription of its target genes but also modulates their RNA splicing. A direct role of SOX proteins in pre-mRNA splicing has been already reported in an *in vitro* assay by Ohe *et al*.^10,11^, who showed that the HMG box of either SOX9 or SRY can restore the splicing activity of SOX6-depleted nuclear extracts. They also demonstrated that SRY and SOX6 were co-localized with splicing factors in the nucleus of mammalian cell lines, consistent with their suggestion that SOX6 acts as a general ‘splicing factor’. They proposed that the splicing activity of the SOX HMG domain might be due to its capacity to bend the pre-mRNAs by affecting their structure and therefore supporting or enhancing protein-RNA and RNA-RNA interactions. In the light of these data, it is tempting to propose as suggested by these authors, that in the amphibian oocyte, SOX9 could affect directly or indirectly the structure of the RNP transcripts through its RNA bending activity and promote RNA-protein and protein-protein interactions, thus, contributing to their post-transcriptional processing.

Little data are available as yet, on the interaction between other SOX proteins such as SOX6 and SRY and RNA. The SOX2 protein was shown to interact with rhabdomyosarcoma 2-associated transcript (RMST), a long noncoding RNA essential for neurogenesis^17^. However, there is mounting evidence from recent investigations^64^ and hypotheses^65^ to support RNA binding for TFs which might interact with nascent RNP complexes to exert downstream functions in RNA regulatory events^65^. A well documented example concerns the transcription factor WT1 (Wilms’ tumor 1)^43,66,67^.

Our results raise the question of whether the binding of SOX9 to RNPs also takes place in the oocyte of other vertebrates. It is noteworthy that oocyte expression of the *Sox9* gene is restricted to the anamniotes clade (fishes and amphibians). No expression has been reported to date in ovarian germ cells of amniotes (reptiles, birds and mammals). In fishes, two *sox9* genes (*sox9a* and *sox99b/a2*) are present in several teleosts [Medaka^33,34,68^; Zebrafish^29,30,69^; Rice field eel^70^]. One paralog is expressed in somatic Sertoli like-cells of the male gonad, similarly to other vertebrates. The second duplicate is expressed in the oocyte. However, almost all of the studies carried out on fishes were performed at the level of mRNA expression only and the presence of the SOX9 protein in the nucleus was not explored. In this context, it would be interesting to investigate the association of SOX9 with the RNP transcripts in the zebrafish oocyte where LBCs have been described^71^. In amphibians, the expression of a single *sox9* gene in the testis and ovary was reported in *X. tropicalis*^38^, *Pleurodeles waltl*^37^ and *Bufo marinus*^36^. In *Rana Rugosa*, a *sox9 alpha* gene and a truncated *beta* isoform have been reported, and both are expressed during gonad development in males and females^40^. In the allotetraploid frog *Xenopus laevis*^72^, two separate *sox9A* and *B* genes are present, *sox9B* being expressed in the gonads of both sexes. It therefore appears that among vertebrates, only the amphibians exhibit a *sox9* gene that displays the dual expression pattern found in teleost fish gonads, i.e., in the somatic cells of the testis and the germ cells of the ovary.

It is widely accepted that the *Sox9* gene has been conserved as a Sertoli-expressed gene in all vertebrate species. However, few hypotheses have been proposed to explain its expression in the oocyte. Until recently, the expression of *Sox9* in the ovary was considered as a fish-specific feature^31^. The teleost fishes underwent an additional round of whole-genome duplication (WGD) as a fish-specific whole-genome duplication event 225 to 333 million years ago^73,74^. It has been suggested that, as a result of this duplication, the zebrafish *sox9a* gene has retained a testis-specific function while *sox9b* acquired a new function in the ovary^29^. Alternatively, the ancestral *Sox9* gene in vertebrates may have been expressed in both the male and female gonads. This pattern would have been conserved until the divergence between the anamniotes, which kept the dual pattern, and the amniotes, which retained a testicular expression only. In this context, it would be interesting to investigate the expression of *soxE3*, the ortholog of the gnathostome *sox9*, in the gonads of lamprey, one of the jawless vertebrate lineages, which diverged from the jawed vertebrates 500 million years ago.

In conclusion, our study demonstrates that the LBCs of the amphibian oocyte and that of *P. waltl* in particular (whose genome has been recently sequenced^75^ provide a powerful *in vivo* system for the further investigation of the RNP-binding capacity of other TFs and a characterization of the protein domains involved because they offer the possibility of a direct and simultaneous visualization of active and inactive chromatin compartments.

## Methods

### Ethical statement

Animal studies were approved (Agreement: N° 01612.01) by the Animal Experimentation Ethical Committee Buffon (CEEA-40) supervised by the French Ministry of Education and Research, and conducted in accordance with the guidelines of the EU directive 2010/63/EU for the housing and care of laboratory animals used for scientific purposes.

### Oocyte collection

*Xenopus laevis* and *Pleurodeles waltl* were raised at 20° and *Xenopus tropicalis* at 25° in separate Techniplast aquariums, illuminated with a photoperiod of 12 h, in the animal housing facility at the Jacques Monod Institute (licence N° B-75-13-17). Ovarian biopsies were performed on adult females that were anesthetized in 0.15% MS222 (Amino-benzoic Acid Ethyl, Fluka). Stage II-VI oocytes were selected and maintained in MBS buffer (Modified Barth’s solution) at 18°, as detailed in^76^. The total number of different females involved was: 14 *P. waltl*; 8*X. laevis*; 6*X. tropicalis*.

### *X. tropicalis sox9* gene amplification and cloning

Total ovary RNA from two *Xenopus tropicalis* females was extracted using Trizol Reagent (Invitrogen, Carlsbad, CA, USA) according to the manufacturer’s instructions. RNA extract was treated with DNase I (Invitrogen, Carlsbad, CA, USA). RNA was reverse transcribed to obtain cDNA using Transcriptor Reverse Transcriptase following manufacturer’s protocols (Lifescience. Roche.com). The open reading frame (ORF) of *X. tropicalis sox9* was amplified from total ovaries cDNA using the following primers FW: ATGATGAATCTCTTGGATCCCTTCATG; Rev:TGGGGCCTGGTGAGCTGTGTATA and PFX Taq polymerase (Invitrogen, Carlsbad, CA, USA) under PCR Touch Down conditions: 30 s denaturation at 94°, 30 s annealing at 65° to 58°, 1.30 mn elongation at 68° (10 cycles) followed by 24 cycles with a 30 s annealing step at 58°. The *sox9* PCR product containing the ORF and the sequence (GATCCAAAAAAGAAGAGAAAGGTAGATCCAAAAAAGAAGAGAAAGGTGGAT) encoding an NLS as control were cloned in the pcDNA3.1/CT-GFP-TOPO^®^ vector (Invitrogen, Carlsbad, CA, USA) following manufacturer’s protocols.

### Cell culture and transient transfections

Cos-7 cells were grown in DMEM F-12 medium supplemented with 10% FBS and 1% penicillin/streptomycin. Cells were seeded 16 h prior to transfection at a density of 2.5 × 10^4^ cells.cm^−2^ and transfected using the calcium-phosphate method with the *X. tropicalis pcDNA sox9-CT-GFP* or the control *pcDNA NLS-CT-GFP* vector. For subcellular localization analysis, cells were fixed 48 hr after transfection in 2% PFA for 30 min at 4°, DNA was stained with 4′,6-diamidino-2-phenylindole dihydrochloride (DAPI) (Merck) (1:1000 for 2 min). Coverslips were mounted on glass slides with DakoCytomaton (Dako). Cells were visualized by epifluorescence microscopy using a Leitz Aristoplan microscope with a Leitz objective lens 6100/1.37 Oil with a numerical aperture of 0.17. Image acquisition was carried out 48 hr after transfection in the 2*X* buffer (Tris 100 mM, pH 6,5, SDS4%, Bromophenol blue 0.2%, glycerol 20%). For western blot analyses, cells were lysed 48 h after transfection in lysis buffer supplemented with 50 mM Tris, 150 mM NaCl, 1 mM EDTA, 1% Triton X-100, pH 7.6, supplemented with protease inhibitors (Complete mini EDTA-free cocktail, Roche) and phosphatase inhibitors (PhosSTOP, Roche). The homogenate was sonicated 1 × 10 min at 4°. After centrifugation (12,000xg for 20 min at 4°), the supernatant was mixed with 5% Beta-mercaptoethanol and heated at 95 °C for 5 min.

### Control of the specifity of the anti-human SOX9 antibodies against the *Xenopus tropicalis* SOX9 protein

Cos-7 cells were transfected with the *pcDNA Xt-sox9-CT-GFP* vector encoding the*X. tropicalis* SOX9 protein (XtSOX9-GFP). As a negative control, cells were transfected with the *pcDNA NLS CT-GFP* vector encoding the NLS-GFP protein, which contains a nuclear localization signal (NLS) that drives GFP translocation to the nucleus. As shown in Figure S2A–B, the newly synthesized XtGFP-tagged SOX9 and NLS-GFP proteins were expressed in the nuclei of mammalian cells. Western blot analyses showed that the Xt GFP-SOX9 protein synthesized in the human cells was recognized by the two polyclonal anti-SOX9 antibodies (Fig. S2C–D).

### RNA synthesis and oocyte microinjection

The *X. tropicalis sox9-CT-GFP Topo TA* and the control *NLS-CT-GFP topo TA* vectors were linearized and used as templates for *in vitro* synthesis of capped and tailed sense-strand RNA using the mMESSAGE mMACHINE^®^ Kit procedure and the Poly (A) Tailing kit (Ambion^TM^). Oocytes were defolliculated for 2–3 hours in saline buffer OR2 containing 0.15% collagenase type II (Sigma Chemical, St. Louis, MO). Fully grown oocytes (stage V-VI) were selected and maintained overnight in MBS buffer (Modified Barth’s solution) at 18°. Ten nanoliters of the RNA solution (1 µg/µl) was injected into the cytoplasm of oocytes at stage V-VI. All injections were performed under a dissection microscope using the nanoject II (Drummond scientific Company) as previously described^46^. After injection, the oocytes were incubated for 24 h at 18° and the newly synthesized GFP-tagged translation products were analyzed by fluorescence microscopy on nuclear spreads or on immunoblots of extracted proteins.

### Oocyte proteins extracts

Two methods were used for the preparation of nuclear extracts^49,77^. In the first one, the GVs were placed in Tris-ethanol solution (6 mmol/L Tris, 45% ethanol, 45% glycerol), right after their extraction from the oocytes to precipitate the nuclear content. A few minutes later, the precipitated nuclear extract became retracted and well detached from the nuclear envelope. The nuclear envelope was then removed and the envelope-free nuclear extracts were stored at −20° in ethanol-glycerol (6 mmol/L Tris, 3 mmol/L MgCl2, 70% ethanol). In the second, oocytes were incubated in MBS containing 2% TCA (W/V) for 20 min at 4° to precipitate the proteins, then washed several times in MBS. GVs were isolated, transferred to microtubes, washed in water, and air-dried. Before electrophoresis, the precipitate was recovered by 5 min centrifugation at 12,000 × g at 4°. Nuclear extracts were homogenized in Laemmli buffer, heated for 10 min at 95°, centrifuged for 5 min at 12,000 × g.

### Western blots

Protein extracts were loaded on 10% or 4–12% NuPage gels (Invitrogen). After transfer, nitrocellulose membranes (Hybond-ECL, GE Healthcare) were incubated overnight in PBS with 0.1% Tween 20 (PBST) and 5% fat-free dry milk at 4°. Membranes were then incubated 1 hr with the primary rabbit polyclonal anti-SOX9 antibodies raised against either the N-terminal (amino acids 1 to 62)^51^ or the C-terminal (amino acids 408 to 504) region of the human SOX9 protein^21^ diluted to 1:400, or with an anti-GFP monoclonal antibody (Lifescience.Roche.com) diluted to 1:1000 or with the monoclonal anti-alpha-tubulin (Sigma) diluted to 1:2000 Membranes were then washed in PBST, incubated with either an HRP conjugated goat anti-rabbit or an HRP conjugated rabbit anti-mouse (Jackson ImmunoResearch) diluted to 1:10,000 in PBS with 0.5% BSA for 1 hr and washed in PBST. Detection was performed using the ECL “SuperSignal West Femto” (Thermo Scientific). Membranes were scanned with the LAS-3000 imaging system (FUJI).

### Nuclear spreads of amphibian oocytes

Stage II-VI oocytes from *P. waltl* were selected and maintained in MBS buffer (Modified Barth’s solution) at 18 °C, as detailed in^76^. Stages V-VI GVs, were manually isolated from *X. tropicalis* and *X. laevis* females in 83 mM KCl, 17 mM NaCl, 6.5 mM NA2HPO4, 3.5 mM KH2PO4, 1.0 mM MgCl2, 1.0 mM Dithiothreitol (DTT)) at pH 7.0 and dissected in a centrifugation chamber filled with dispersal medium (20.7 mM KCl, 4.3 mM NaCl, 1.6 mM NA2HPO4, 0.9 mM KH2PO4, 1.0 mM MgCl2, 1.0 mM Dithiothreitol (DTT), 0.1% w/v paraformaldehyde) at pH 6.6–6.8 for *X*. l*aevis* or pH 7.0 for *X. tropicalis*^76,78,79^. Germinal vesicles were manually isolated from stage II to VI oocytes of *P. Waltl*, and dissected in 75 mM KCl, 25 mM NaCl, 0.01 mM MgCl2 and 0.01 mM CaCl_2_, pH7.2 and LBCs were prepared as previously described^60^. Nuclear spread preparations were centrifugated at 300×g for 10 min, and at 3,000×g for 30 min at 4° before processing for immunostaining.

### Transcription inhibition

*P. waltl* oocytes were incubated for 24 h in MBS Buffer supplemented or not (controls) with either Actinomycin D (5μg/ml, Sigma) or alpha-amanitin (20μg/ml, a gift of O. Bensaude). LBCs spreads were prepared as previously described.

### RNase treatment

Nuclear spreads of *X. laevis* were prepared as previously described. They were then digested 30 min at 37° with RNase A (Roche Life Science, France) (20μg/ml). After washing 3 × 10 min in PBS, preparations were processed for immunostaining experiments as described below.

### Immunostaining of nuclear spreads

Nuclear spread preparations were fixed for 30 min at 4° in phosphate buffer saline (PBS) containing 2% paraformaldehyde, washed 3 × 10 mn in PBS and blocked for 10 mn with Horse serum at 10% dilution. They were then incubated with the relevant primary antibody (see below) for 1 hr at room temperature, washed 3 × 10 mn with PBS, incubated with the secondary antibody for 1 hr at room temperature, washed again in PBS for 1 hr. Preparations were post-stained with Hoechst 33342 (1:1,000; Invitrogen) and mounted using Citifluor (Biovalley). The two rabbit polyclonal antibodies raised against N-terminal (amino acids 1 to 62) (Nter-SOX9 Ab)^51^ or C-terminal (amino acids 408 to 504) regions of the human SOX9 protein(Cter-SOX9 Ab)^21^ were used at 1:100 and 1:400 dilution, respectively.The anti-coilin antibody (H1) (Santa Cruz Biotechnology) was used at 1:200 dilution. The mouse IgM anti-RNA pol II CTD phospho-epitope (mAb H14) and the mouse IgG anti-CELF1 (mAb 3B1) (kindly supplied by Dr. J. Gall) were used at 1:5000 and 1 μg/ml dilution, respectively. The mouse anti-SR antibody (1H4) raised against *X. laevis* SR proteins (Santa Cruz Biotechnology) was used at 1:200 dilution. The secondary antibodies Alexa fluor 488, Alexa fluor 568 IgG, goat anti-rabbit, Alexa fluor 568 IgG goat anti-mouse (Invitrogen Corp., Carlsbad, CA) or Alexa 488 goat anti-mouse IgM (Life technlogies) were used at 1:1000 dilution. Standard transmitted light and fluorescence microscopy were performed using a wide field Leica microscope with a plan Apo 100*X* oil objective (NA = 1.4). Images were captured using a CoolSnap HQ, Photometrics camera driven by the software Metamorph6 (Universal imaging). In order to probe accurately the fluorescence, series of confocal Z-planes (0.7 µm distance) were collected. Pixel size of the image was 64.5 nm. Structured illumination microscopy was using an ELYRA PS.1 Zeiss microscope. Structured illumination was achieved using appropriate grids for the different excitation wavelengths (405 nm, 488 nm and 561 nm).For each plane three grid rotations and 5 different phases were taken. A Zeiss PLAN-APO 63x NA 1.40 objective was used. The microscope was coupled to an EMCCD camera (Andor iXon 885). For each series of experiments, image acquisition was conducted using the same settings. The intensity of fluorescence in the different experiments was normalized with respect to their relevant controls with the secondary antibodies Alexa fluor alone.

### Quantification of fluorescence density and colocalization

A specific algorithm for image processing was developed to quantify fluorescence density of each chromosome using the ImageJ software. Chromatin areas were first isolated on the Hoechst images using an automatic thresholding with the Otsu’s method after removing noise with a filter and background correction. The resulting region of interest (R.O.I) designated as MASK was then applied to the GFP images. Fluorescence density was quantified by measuring the average intensity of the GFP images inside masks previously built (Fig. S6). Colocalization analyses were carried out using the ImageJ plugin “Coloc2”.

### Statistics

Statistical significance for mean values was estimated by Student *t*-Tests using Microsoft’s Excell. Error bars represent the standard deviations. The degree of colocalization between SOX9 and CELF1 granules was estimated using a Pearson correlation coefficient over a large number of pixels.

## Electronic supplementary material

Supplementary information
