# Supplementary material for: A role for SOX9 in post-transcriptional processes: insights from the amphibian oocyte
Source: Sci Rep. 2018 May 8;8:7191. doi: 10.1038/s41598-018-25356-1 (PMC5940923; doi:10.1038/s41598-018-25356-1)
Supplement: Supplementary file 1 — Supplementary information [file 41598_2018_25356_MOESM1_ESM.pdf]

# Supplementary information

## A role for SOX9 in post-transcriptional processes: insights from the amphibian oocyte

M. Penrad-Mobayed[1*], C. Perrin[1], D. L'Hôte[2], V. Contremoulins[1], J.-A. Lepesant[1],

B. Boizet-Bonhoure[3], F. Poulat[3], X. Baudin[1], R. A. Veitia[1].

1. Institut Jacques Monod. CNRS UMR 7592, Université Paris-Diderot, Paris, France

2. Unité de Biologie Fonctionnelle et Adaptative (BFA). CNRS UMR 8251, Université Paris- Diderot, Paris, France

3. Institut de Génétique Humaine. CNRS UMR 9002, Montpellier, France

(*) Corresponding author: may.penrad@ijm.fr

Institut Jacques Monod. CNRS UMR 7592, Université Paris-Diderot. 15 rue Hélène Brion. 75205 Paris Cedex13. France.

Runnning title:  A role for SOX9 in post-transcriptional processes?

```
N-terminal region of SOX9 (amino acids 1-62)

hSOX9    MNLLDPFMKM TDEQEKGLSG APSPTMSEDS AGSPCPSGSG
Xt SOX9  MNLLDPFMKM TEEQDKCMSG APSPTMSEDS AGSPCPSGSG
Xl SOX9A MNLLDPFMKM TEEQDKCMSG APSPTMSDDS AGSPCPSGSG
Xl SOX9B MNLLDPFMKM SEEQDKCLSG APSPTMSEDS AGSPCPSGSG
Pw SOX9  MNLLDPFMKM TEEQEKCLSG APSPSMSEDS VGSPCPSGSS
         ********** +++*+*  ** ****+**+** +********

hSOX9    SDTENTRPQE NTFPKGE-PDL KK
Xt SOX9  SDTENTRPQE NTFPKGD-PEL KK
Xl SOX9A SDTENTRPQE NTFPKGD-QEL KK
Xl SOX9B SDTENTRPQE NTFPKGD-QEM KK
Pw SOX9  SDAENTRPME NGFPKGELQEL KK
         ** ***** * * ****+  +  **


C-terminal region of SOX9 (amino acids 408-504)

hSOX9    EQ QQHSPQQIAY SPFNLPHYSP SYPPITRSQY DYTDHQNSSS
Xt SOX9  QQ QQHSPQQLNY SSFNLQHYSS SYPTITRAQY DYTEHQGSNS
Xl SOX9A QQ QQHSPQQLNY SSFNLQHYSS SYPTITRAQY DYTEHQGSST
Xl SOX9B QQ QQHSPQQLNY SSFNLQHYSS SYPTITRAQY DYTEHQGSNS
Pw SOX9  EQ QQHSPQQLSY SPFNLQHYNS TYPTITRAQY DYTDHQSSNT
          * *******+ * * *** **   +** *** ** ***+** * +

hSOX9    YYSHAAGQGT GLYSTFTYMN PAQRPMYTPI ADTSGVPSIP
Xt SOX9  YYSHASGQNS GLYSNFSYMN PSQRPMYTPI ADTTGVPSIP
Xl SOX9A YYSHASGQNS GLYSTFSYMN PSQRPLYTPI ADTTGVPSIP
Xl SOX9B YYTHASGQNS GLYSNFTYMN PSQRPMYTPI ADTTGVPSIP
Pw SOX9  YYSHAAGQSS NLYSTFSYMN PTQRPMYTPI ADTTGVPSIP
         **+** ** +  *** *+*** * *** **** *** ******

hSOX9    QTHSPQHWEQ PVYT
Xt SOX9  QTHSPQHWEQ PVYT
Xl SOX9A QTHSPQHWEQ PVYT
Xl SOX9B QTHSPQHWEQ PVYT
Pw SOX9  QTHSPQHWEQ PVYT
         ********** ****
```

Figure S1: Comparison of amino acid sequences of the N-terminal and C-terminal regions of the human SOX9 protein with those of *X. tropicalis* (Xt), X. *laevis* (Xl) and *P. waltl* (Pw)

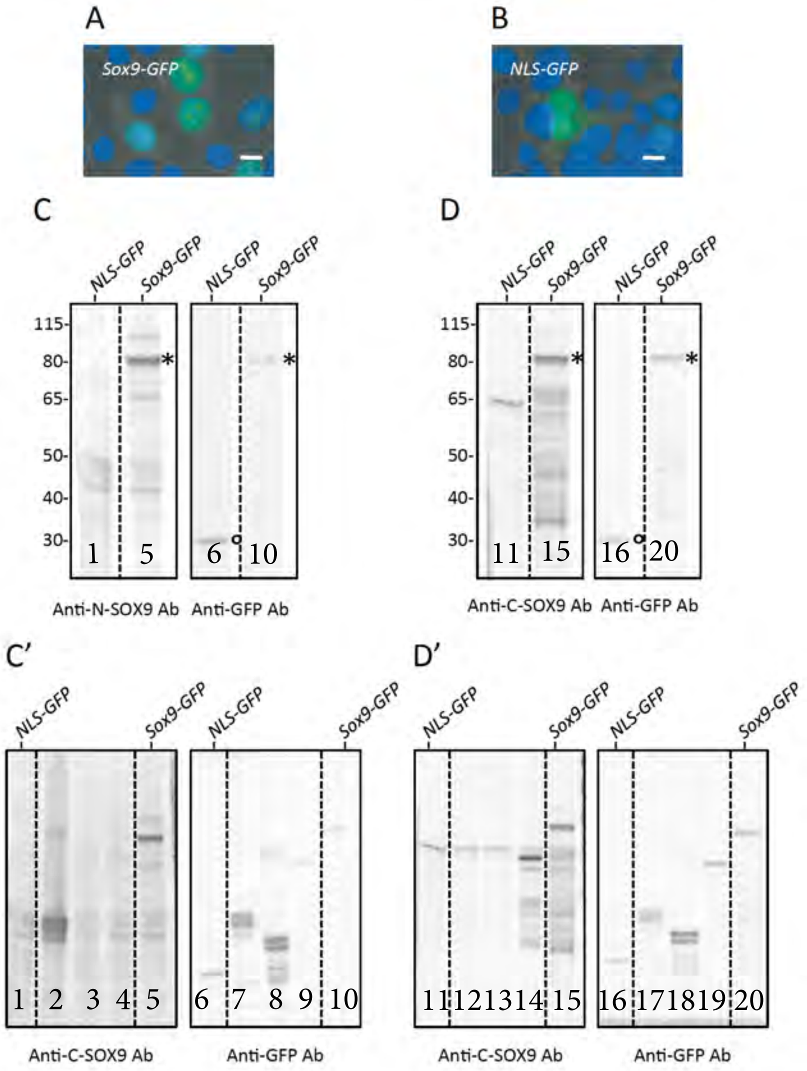


Figure S2**:** Control of the specificity of the anti-human SOX9 antibodies against the *Xenopus tropicalis* SOX9 protein. COS-7 cells were transfected with the pcDNA *Xt-sox9 CT-GFP* (*Sox9-GFP*) or the *pcDNA NLS-CT-GFP* (*NLS-GFP*) vector as a negative control. A, B: Subcellular localization of the GFP-tagged proteins. The SOX9-GFP(A) and NLS-GFP (B) proteins were detected in the nucleus. C, D: Western blots analysis of protein extracts of transfected cells with *sox9-GFP* or the control *NLS-GFP* vectors. The expression of the GFP-tagged proteins was monitored using three antibodies against the N-terminal (anti-N-SOX9 Ab) or C-terminal regions of the human protein (anti-C-SOX9 Ab), and a mouse monoclonal anti-GFP antibody (anti-GFP Ab). The dashed lines separate the lanes which were cut out the original immunoblots in ( C', D'). Note that a polypeptide ca. 80 kDa (*) that corresponded to the fusion protein was detected in the protein extracts of cells transfected with the *Sox9-GFP* vector using both the polyclonal anti-human SOX9 antibodies and the anti-GFP antibody. In cells transfected with the *NLS-GFP* vector, a 30 kDa band (°) corresponding to the molecular mass of the GFP protein was detected with the anti-GFP antibody.

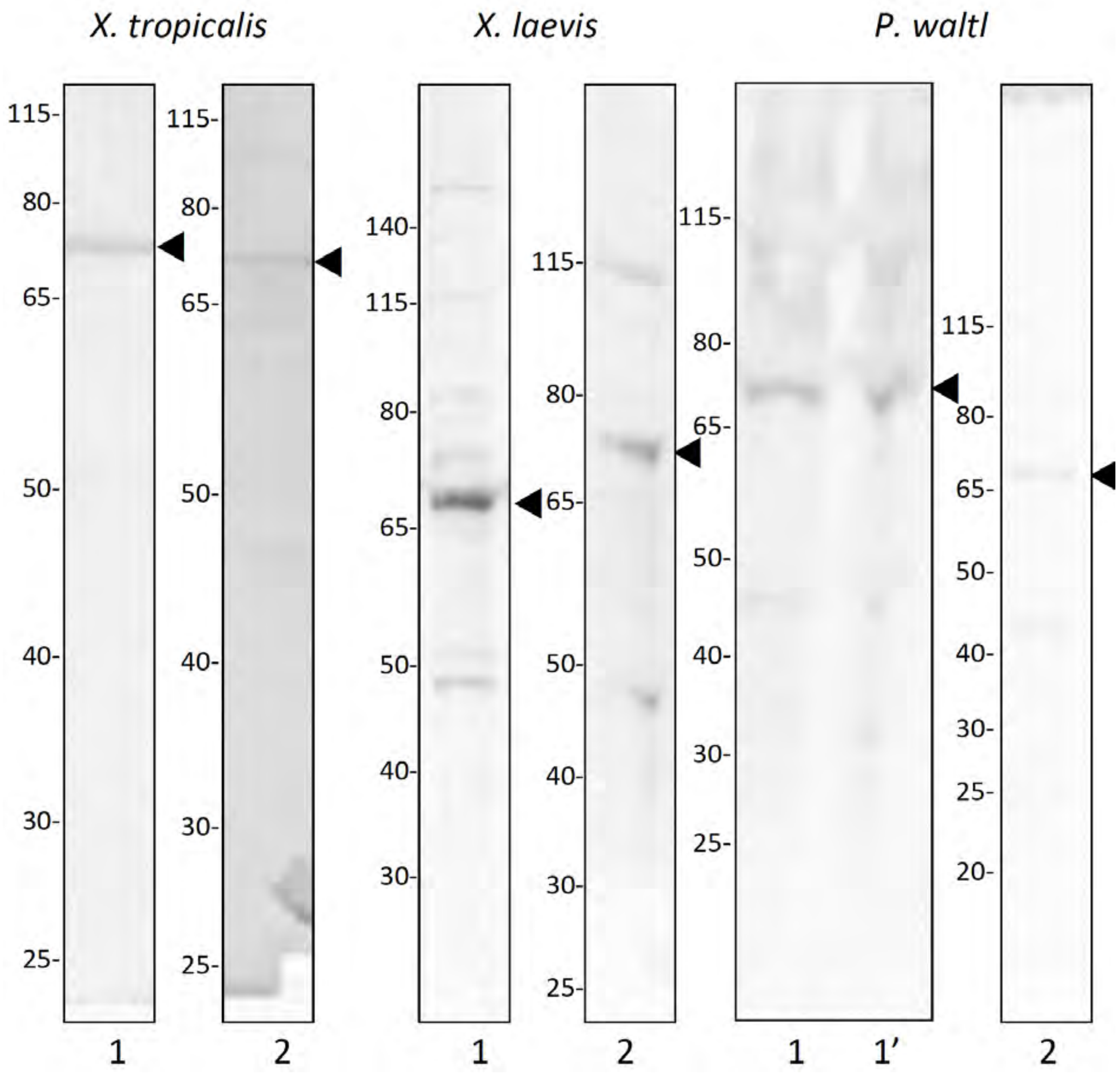


Figure S3 **:** Expression of the SOX9 protein in the oocyte of *X. tropicalis*, *X. laevis* and *P. walt*. Western blot analysis of SOX9 in protein extracts (30 GVs equivalents per lane for *X. tropicalis*, 20 for *X. laevis* and 15 for *P. waltl*) using the two anti-human SOX9 antibodies: anti-Nter (Lanes ‘1’) or anti-Cter (lanes ‘2’). The *P. walt* panel (I’) corresponded to a nuclear extract from 15 GVs whose nuclear envelope had been removed. The antibodies recognized a major polypeptide around 68 kDa in the three species.

A

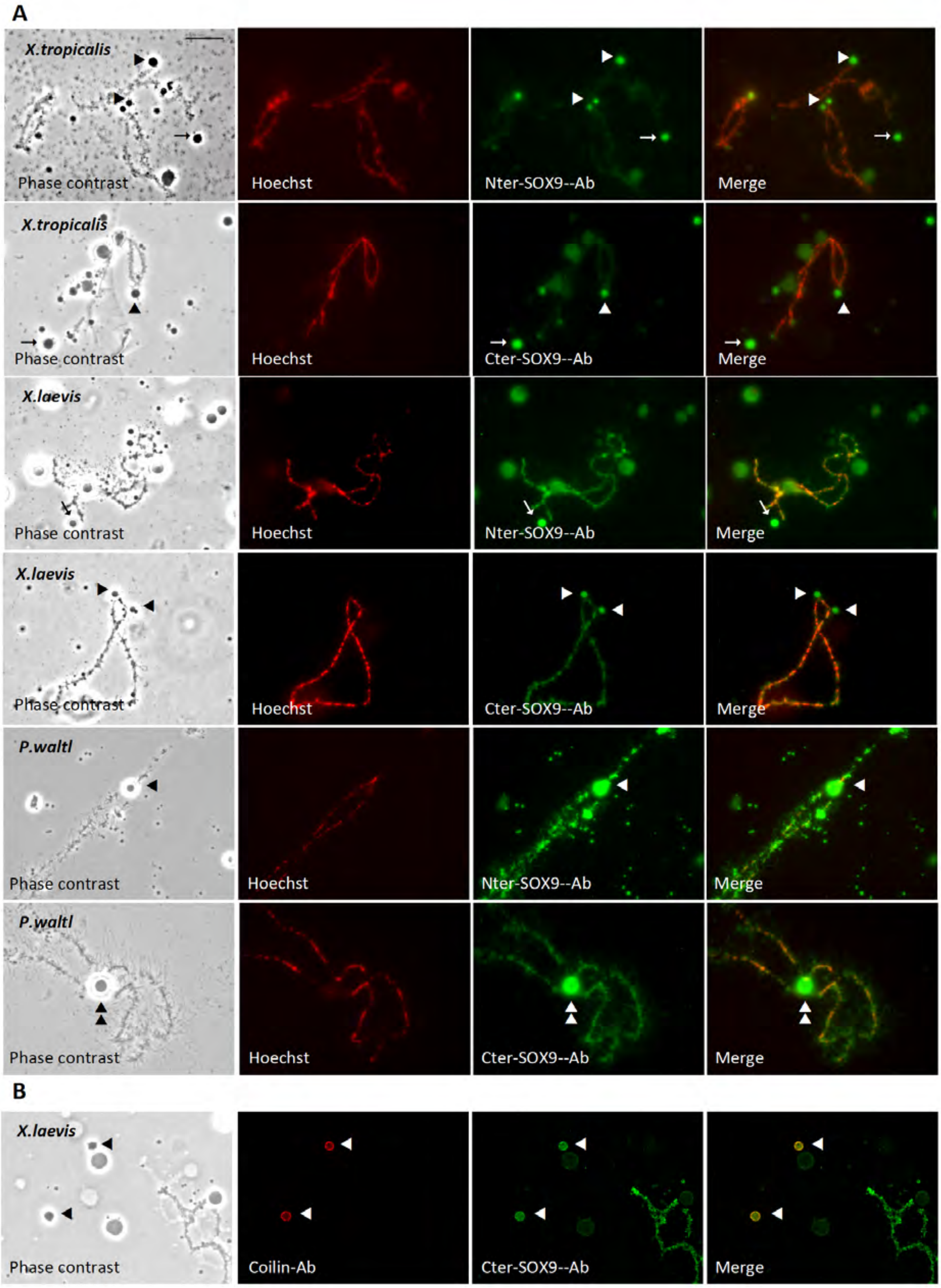


Figure S4: Subnuclear localization of SOX9 in the GV of *X. tropicalis, X. laevis and P. walt*. Immunostaining of nuclear spreads using the two anti-SOX9 antibodies (Nter-SOX9-Ab and Cter-SOX9-Ab) (green, Alexa 488 IgG) and counterstained with

Hoechst (red). A: the Nter-SOX9-Ab and Cter-SOX9-Ab stained the LBCs. They also stained nuclear bodies either attached to the LBCs (arrowheads) and the Histone Locus Body (double arrowheads) or free in the nucleoplasm (arrows). Note that the size of the LBCs of *P. waltl* is much larger than that of *X. laevis* and *X. tropicalis.* B : Co-staining of SOX9 (green, Alexa 488 IgG) and coilin (Alexa 568 IgG) in the Cajal bodies (CBs) of *X. laevis* identified using the monoclonal anti-coilin antibody (H1). SOX9 co-localized with coilin (arrowheads). Note that the nucleoli were not stained by Cter-SOX9-Ab or coilin-Ab. The intensity of fluorescence was normalized with respect to the relevant controls with the secondary antibodies alone. Wide field Leica microscope. Scale bar for all micrographs: 15 μm.

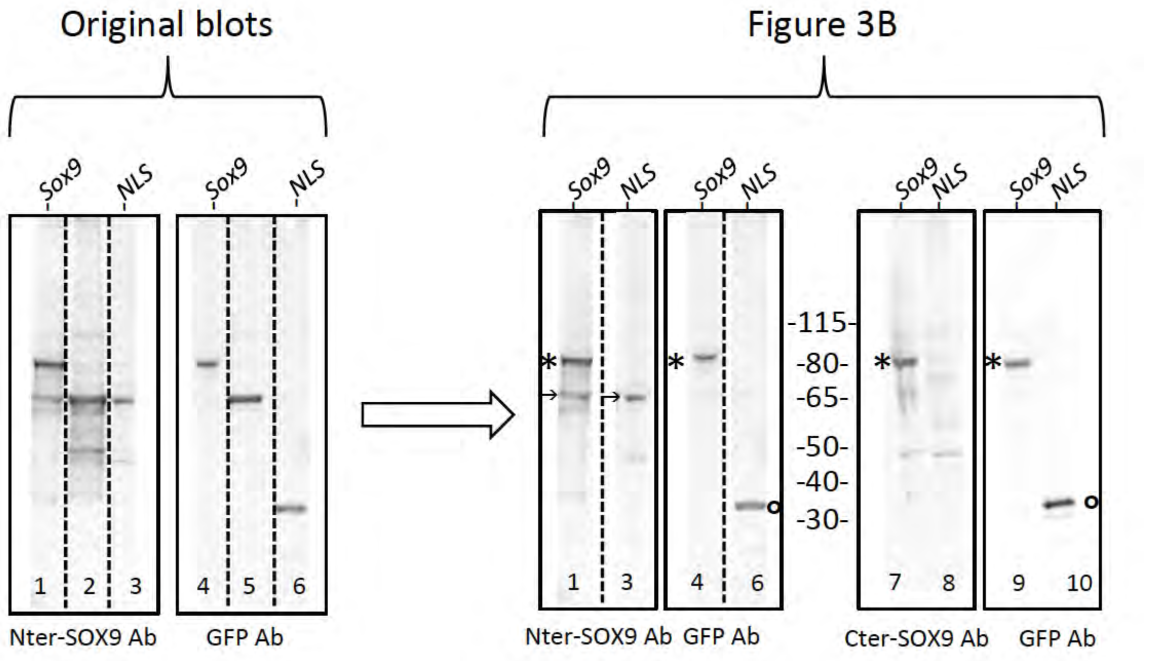


Figure S5 **:** Left panel : original immunoblots (Nter-SOX Ab or GFP Ab) from which lanes 1, 3, 4, 6 were cut out to mount figure 3B (dashed lines). Right panel : Immunoblots of nuclear extracts from injected oocytes with the *sox9-CT-GFP* (*Sox9*) or *NLS-CT-GFP* (*NLS*) transcripts. Each lane corresponds to 15 GVs. Molecular weight markers are indicated. A polypeptide (*) ca. 80 kDa detected with the anti-Nter or Cter- SOX9 antibodies (lanes 1 and 7) and the anti-GFP antibody (lanes 4 and 9) in the *(Sox9)* injected oocytes, corresponded to the Sox9-GFP protein. In the *(NLS)* injected oocytes, a ca. 30kDa polypeptide (°) corresponding the NLS-GFP protein (lanes 6 and 10) was detected with the anti-GFP antibody. The polypeptide of ca. 68 kDa molecular weight (arrows in lanes 1, and 3) corresponded to the endogenous SOX9 protein. Lane 3 is the same as lane1 (*X. laevis*) shown in figure S3.

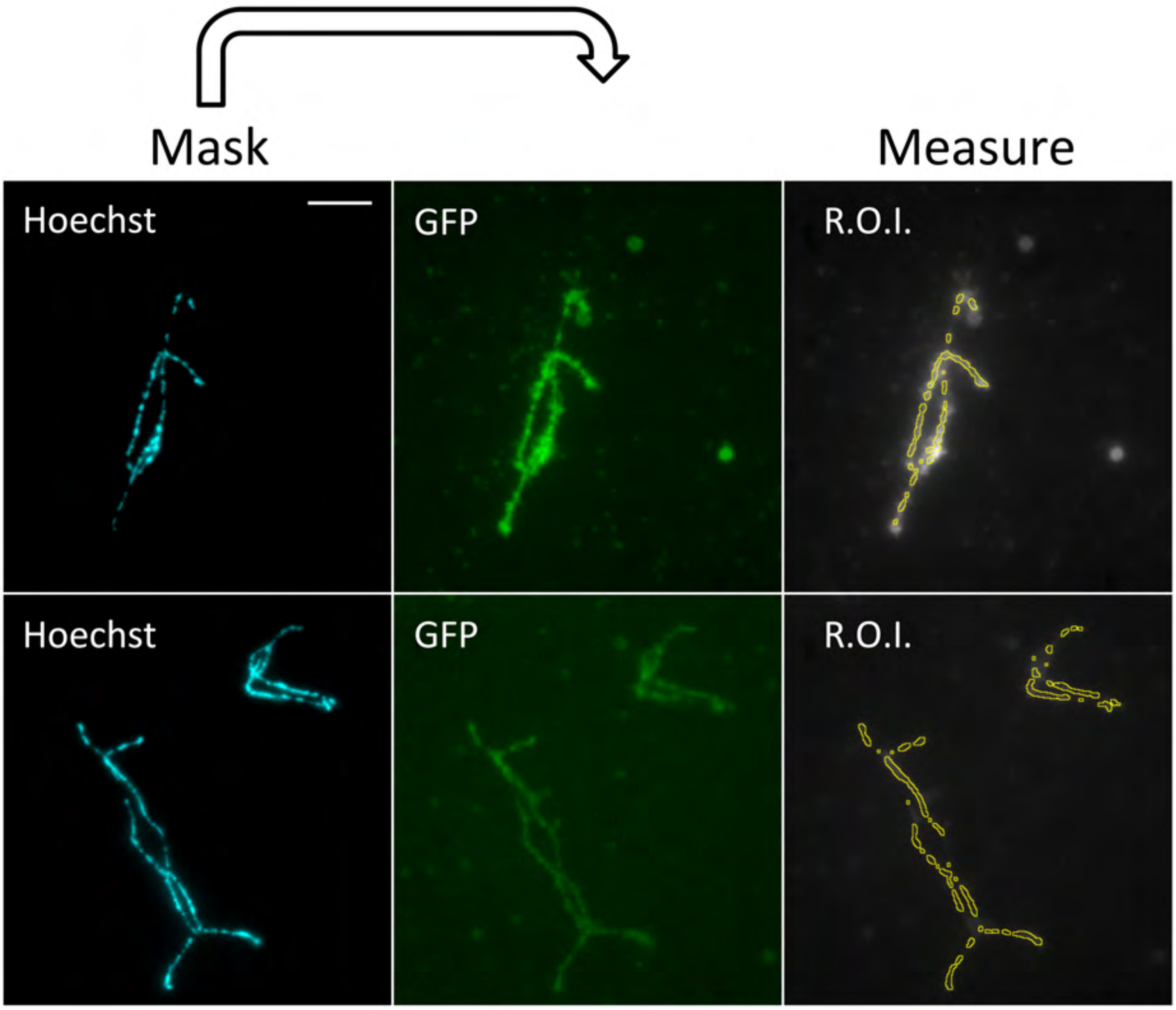


Figure S6: Principle of the quantification of fluorescence density of Xt-SOX9-GFP and NLS-GFP in LBCS of *X. laevis*. A specific algorithm for image processing was developed to quantify fluorescence density of each chromosome using the ImageJ software. Chromatin areas were first isolated on the Hoechst images using an automatic thresholding with the Otsu's method after removing noise with a filter and background correction. The resulting region of interest (R.O.I) designated as MASK was then applied to the GFP images. Fluorescence density was quantified on the R.O.I by measuring the average intensity of the GFP images inside masks previously built.

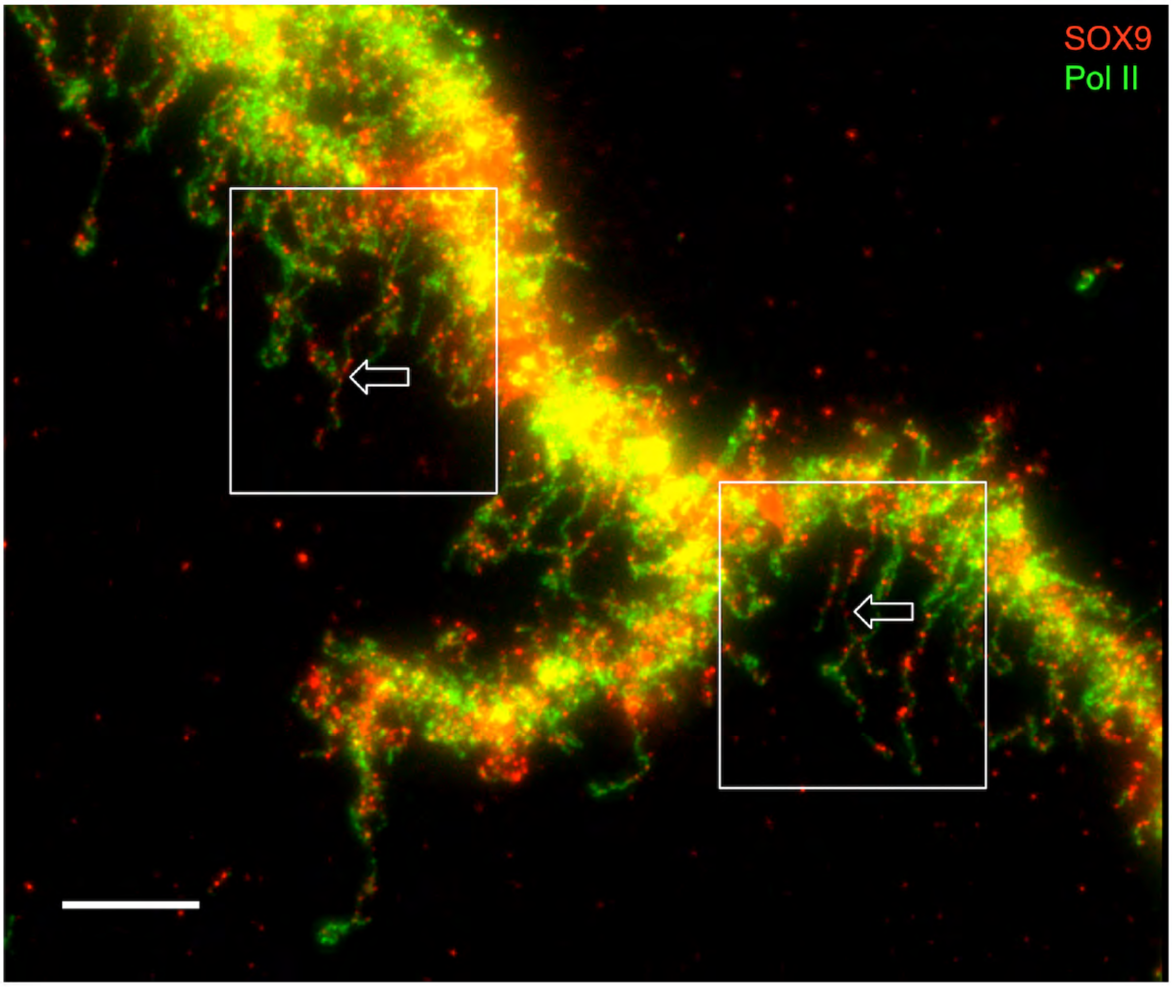


Figure S7: (corresponds to the non-deconvoluted Figure 5). Immunostaining of Pol II and SOX9 in *P. waltl* LBCs. Nuclear spreads were immunostained for SOX9 with the anti-Cter-SOX9 antibody (red, Alexa 568 IgG) and for Pol II with the mAbH14 (green, Alexa 488 IgM). Pol II immunostaining was continuous over the loop axis while that of SOX9 was concentrated on granules distributed above the loop axis. These double immunostaining patterns were particularly visible on the two lateral loops indicated by arrows in the boxed areas. The intensity of fluorescence in the different experiments was normalized with respect to their relevant controls with the secondary antibodies alone. Wide field Leica microscope. Scale bar: 10 μm.

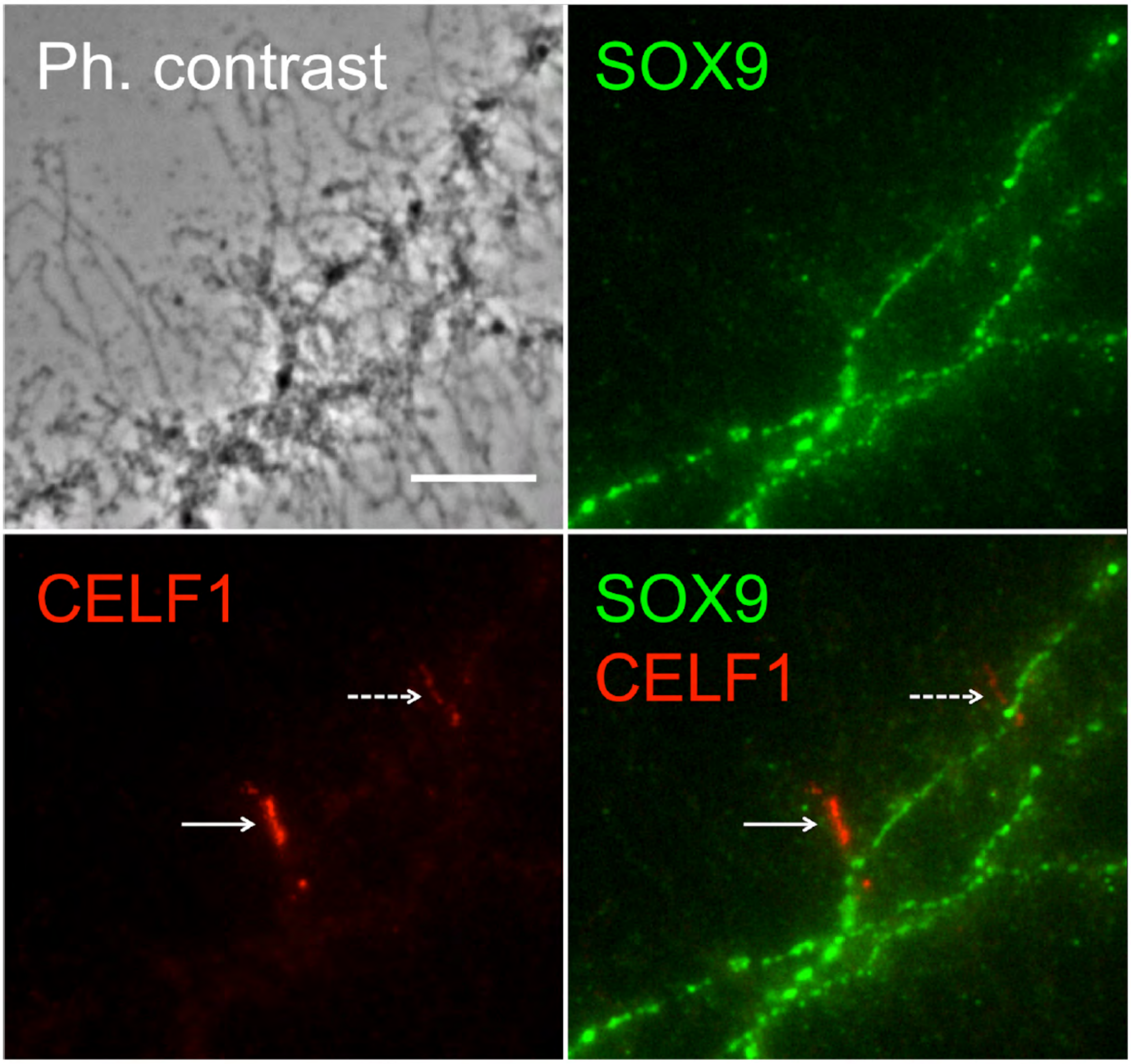


Figure S8: Binding of CELF1 to a subset of lateral loops of *P. waltl*. Nuclear spreads were immunostained for SOX9 with the anti-Cter-SOX9 antibody (green, Alexa 488 IgG) and for CELF1 with mAb3B1 (red, Alexa 568 IgG). The mAb3B1 immunostained very few loops by comparison with the anti-Cter-SOX9 antibody which immunostained most of lateral loops. The solid-line arrow points to a strongly labeled loop while the dotted arrow points to a weakly labeled loop. The position of these loops is indicated in the matching phase contrast images. The intensity of fluorescence in the different experiments was normalized with respect to their relevant controls with the secondary antibodies alone. Wide field Leica microscopy. Scale bar: 10 μm.
